# Modeling the Potential Impact of Missing Race and Ethnicity Data in Infectious Disease Surveillance Systems on Disparity Measures: Scenario Analysis of Different Imputation Strategies

**DOI:** 10.2196/38037

**Published:** 2022-11-09

**Authors:** Bahareh Ansari, Rachel Hart-Malloy, Eli S Rosenberg, Monica Trigg, Erika G Martin

**Affiliations:** 1 Center for Policy Research Rockefeller College of Public Affairs and Policy University at Albany Albany, NY United States; 2 Center for Collaborative HIV Research in Practice and Policy School of Public Health University at Albany Albany, NY United States; 3 New York State Department of Health Albany, NY United States; 4 Department of Epidemiology and Biostatistics School of Public Health University at Albany Albany, NY United States; 5 Department of Epidemiology Rollins School of Public Health Emory University Atlanta, GA United States; 6 Department of Public Administration and Policy Rockefeller College of Public Affairs and Policy University at Albany Albany, NY United States

**Keywords:** missing data, sexually transmitted diseases, imputation, surveillance, health equity

## Abstract

**Background:**

Monitoring progress toward population health equity goals requires developing robust disparity indicators. However, surveillance data gaps that result in undercounting racial and ethnic minority groups might influence the observed disparity measures.

**Objective:**

This study aimed to assess the impact of missing race and ethnicity data in surveillance systems on disparity measures.

**Methods:**

We explored variations in missing race and ethnicity information in reported annual chlamydia and gonorrhea diagnoses in the United States from 2007 to 2018 by state, year, reported sex, and infection. For diagnoses with incomplete demographic information in 2018, we estimated disparity measures (relative rate ratio and rate difference) with 5 imputation scenarios compared with the base case (no adjustments). The 5 scenarios used the racial and ethnic distribution of chlamydia or gonorrhea diagnoses in the same state, chlamydia or gonorrhea diagnoses in neighboring states, chlamydia or gonorrhea diagnoses within the geographic region, HIV diagnoses, and syphilis diagnoses.

**Results:**

In 2018, a total of 31.93% (560,551/1,755,510) of chlamydia and 22.11% (128,790/582,475) of gonorrhea diagnoses had missing race and ethnicity information. Missingness differed by infection type but not by reported sex. Missing race and ethnicity information varied widely across states and times (range across state-years: from 0.0% to 96.2%). The rate ratio remained similar in the imputation scenarios, although the rate difference differed nationally and in some states.

**Conclusions:**

We found that missing race and ethnicity information affects measured disparities, which is important to consider when interpreting disparity metrics. Addressing missing information in surveillance systems requires system-level solutions, such as collecting more complete laboratory data, improving the linkage of data systems, and designing more efficient data collection procedures. As a short-term solution, local public health agencies can adapt these imputation scenarios to their aggregate data to adjust surveillance data for use in population indicators of health equity.

## Introduction

### Background

Infectious disease surveillance systems are important information technologies used to identify outbreaks of infectious diseases, describe the current burden of the diseases, and monitor trends and disparities among populations [1]. However, many surveillance systems have data quality issues [2-4] that must be understood for the correct interpretation of data. Although informatics solutions exist for dealing with data quality issues in surveillance systems [3,5], the optimal solution for a specific surveillance system requires a deeper understanding of the contributing factors and the consequences of data quality issues in interpreting surveillance data. In this study, we focused on missing race and ethnicity information in surveillance systems and explored the effect of missingness on the calculated disparity measures to guide future informatics solutions.

We focused on health equity because racial and ethnic minority populations in the United States continue to experience a disproportionately high burden of poor health outcomes. These disparities can be attributed to persistent systemic racism against African American people in health care settings and medical research throughout the US history [6] and a range of social and structural factors such as residential segregation, lower opportunities for education, unemployment, and lower income [7]. Robust measures of population health using high-quality data are needed for a complete understanding of disparities in health outcomes [8]. Moreover, the data should be representative of the population without coverage bias. A systematic undercounting of communities of color in surveillance data [9], one type of coverage bias, is an example of systematic racism built into government databases, which may skew public health decision-making.

Public health surveillance systems are critical sources of information for measuring and monitoring disparities and evaluating public health initiatives to improve equity [10]. However, incomplete information on race and ethnicity may affect disparity measures. Missing race and ethnicity information has been a major limitation in different health care databases, such as birth certificate records in a large US health care system [11], Veterans’ health administration records [12], reported COVID-19 cases, and persons who received COVID-19 vaccinations in the United States [13]. A previous study found that incomplete race and ethnicity information in COVID-19 data resulted in an underrepresentation of disparities among racial and ethnic population groups [9]. The use of biased disparity measures in policy and funding decisions can perpetuate the legacy of systemic racism.

### Objectives

We examined missing race and ethnicity information in chlamydia and gonorrhea surveillance data from 2007 to 2018 and used 5 imputation strategies to explore how missing demographic information could have impacted our measurement of racial and ethnic disparities. We chose chlamydia and gonorrhea for our exploration because they are among the most common notifiable conditions in the United States [14] and had an estimated total lifetime cost of US $1.0 billion in 2018 [15], and it is well established that non-Hispanic Black and Hispanic populations have persistently higher rates of diagnosed sexually transmitted infections (STIs) than White populations [16]. Our findings highlight the importance of understanding and addressing missing demographic data in surveillance systems to reduce systematic biases in the measures of racial and ethnic disparities.

## Methods

### Study Population and Data Sources

We conducted 2 sets of analyses: (1) a descriptive trend analysis to investigate the extent of missing race and ethnicity information across the 2 infections by reported sex (hereafter, sex) and year and (2) a scenario analysis to assess how the rate ratios (RRs) and rate differences (RDs) changed under different methods to redistribute diagnoses with incomplete demographic data to specific racial and ethnic groups. The study population differed in the descriptive trend analysis and the scenario analysis. For the descriptive trend analysis, we used aggregated state-level counts of all reported chlamydia and gonorrhea cases among male and female patients aged ≥15 years for 50 states and the District of Columbia from 2007 to 2018 (n=612 state-year observations for each infection in male or female patients). For the scenario analysis, we restricted the analysis to 2018 (n=51 state-level observations).

The counts of chlamydia and gonorrhea diagnoses were obtained from the Centers for Disease Control and Prevention’s (CDC) National Center for HIV Viral Hepatitis, STD, and TB Prevention AtlasPlus [17]. The underlying data are from the National Notifiable Disease Surveillance System, a complex surveillance system that is a collaboration among numerous local, state, and federal partners. Gonorrhea and chlamydia are reportable and nationally notifiable conditions. As such, states and territories have set requirements for laboratories and medical providers to report case information to public health departments. In turn, states voluntarily transmit case report data to the CDC, which secures and processes deidentified data that are then provided to disease-specific programs across the CDC [18,19]. This process is complex for several reasons. First, jurisdictions use various surveillance information systems [20]. Adding to the complexity of data collection is that not all newly identified cases are contacted by disease intervention specialists; jurisdictions follow state and federal guidelines regarding which STIs to prioritize for partner services. Chlamydia and gonorrhea cases generally receive a lower priority for follow-up than HIV and syphilis cases [21], which may lead to missing demographic and other information if the surveillance record is based exclusively on laboratory data that are automatically sent to the public health authority without an accompanying case report from the provider.

To establish rates and disparity measures, we used the 5-year American Community Survey 2018 [22] to determine the population in the United States by state, sex, and race and ethnicity. We limited our analysis to non-Hispanic Black, Hispanic, and non-Hispanic White persons because other racial and ethnic groups, including persons with multiple races, had small numbers of reported cases (59,687/1,755,510, 3.4% and 22,134/582,475, 3.8% of the total reported cases for chlamydia and gonorrhea during 2018, respectively). Although these other racial and ethnic groups are important, their small counts impeded our ability to produce stable rates and disparity measures. Male versus female sex was defined as a binary variable, which might represent sex at birth or current identity, as current gender identity is not systematically recorded in the surveillance data.

### Ethical Considerations

The data used in this study were publicly available for direct download from the CDC in an aggregate and anonymized format without use restrictions (ie, number of cases per state by stratum). The granularity of the strata renders it impossible to reidentify the respondents. We did not need to seek a review from our Human Subjects Committee because the nature of the data and the research question were not considered human subjects research by University at Albany policy guidance.

### Statistical Methods

The statistical methods had 4 parts. First, we conducted a descriptive trend analysis of the percentage of diagnoses with unknown race and ethnicity information for chlamydia and gonorrhea in male and female patients in each state from 2007 to 2018. This analysis produced descriptive statistics to explore variations by state, year, sex, and infection, and the Cochran-Armitage test [23] was used to explore the trends of chlamydia and gonorrhea among male and female patients. Second, we calculated the rates and 2 disparity measures based on the available demographic information. To measure racial and ethnic disparities, we chose both RR and RD, following best practices for reporting disparities using multiple measures [21]. Third, we redistributed the diagnoses with unknown race and ethnicity information in 2018 using 5 imputation scenarios. Fourth, we compared the disparity measures under different scenarios with the base case (disparity measures calculated using only available data and no adjustment for missing data) to evaluate the potential impact of missing data. Weights were not applied in the analyses because AtlasPlus provides the total number of known reported cases (ie, the full population) rather than a sample of cases.

Table 1 summarizes the 5 scenarios. The first scenario (scenario 1) was redistributed according to the reported chlamydia and gonorrhea diagnoses with known race and ethnicity information in the same state. We used 2 other methodologies that used available demographic data for chlamydia and gonorrhea diagnoses and redistributed diagnoses with unknown race and ethnicity to population groups based on known diagnoses in neighboring states (scenario 2) or the same region (scenario 3). Neighboring and regional data have been used in previous studies to impute aggregate-level spatial data [24]. Our fourth and fifth scenarios were based on available demographic information from HIV and syphilis in the same state in the same year (2018). These are 2 other common STIs with more complete racial and ethnic information because people with newly reported diagnoses of HIV and syphilis are prioritized for follow-up by disease intervention specialists as part of partner services programs for HIV and STI [25].

For the fourth and fifth scenarios, the racial and ethnic distributions of all 4 infections were not identical. For example, the number of chlamydia and gonorrhea diagnoses is larger among female patients than male patients, whereas the number of HIV and syphilis cases is larger among male than among female patients. However, HIV and syphilis surveillance data are commonly under the purview of surveillance staff and are likely to be accessible to data analysts who calculate disparity measures. Therefore, we added these scenarios as alternative methods for considering the impact of missing race and ethnicity information.

To measure disparities, we used both an absolute measure (RD) and relative measure (RR). Finally, we created visualizations to compare disparity measures produced in each scenario to the base case in which diagnoses with missing race and ethnicity information were excluded from the calculations.

**Table 1:** Summary of imputation scenarios to assign race and ethnicity to reported diagnoses with incomplete demographic information.

Scenario^a^	Description
Base case	No adjustments. This scenario includes reported diagnoses with available race and ethnicity information in National Center for HIV, Viral Hepatitis, STD, and TB Prevention (NCHHSTP) AtlasPlus. Counts of diagnoses with missing race and ethnicity information are omitted from analysis.
Scenario 1	Reallocation based on reported chlamydia and gonorrhea diagnoses with known race and ethnicity from the same state. Within a state, the diagnoses with missing race and ethnicity information are reapportioned to a racial and ethnic group based on their distribution among known diagnoses. For example, if 50% of diagnoses have missing race and ethnicity information and among the remaining diagnoses, 40%, 20%, and 40% are recorded as Black, Hispanic, or White race and ethnicity, then the unknown diagnoses will be reassigned following the 40%-20%-40% distribution. This will not change the *distribution* of cases in terms of the percentage in each racial and ethnic group, but it does increase the *number* of diagnoses within each group.
Scenario 2	Reallocation based on reported chlamydia and gonorrhea diagnoses with known race and ethnicity from neighboring states. Within a state, the diagnoses with missing race and ethnicity information are reapportioned to a racial and ethnic group based on the distribution of known diagnoses in the states that share a contiguous border. In the case of Alaska and Hawaii, which do not have any neighboring states, this scenario does not adjust the rate.
Scenario 3	Reallocation based on information from states in the geographic region. Within a state, the diagnoses with missing race and ethnicity information are reapportioned to a racial and ethnic group based on their distribution in all states within the 4-level US Census region (Northeast, Midwest, South, and West).
Scenario 4	Reallocation based on information from HIV diagnoses within a state. Within a state, diagnoses with missing race and ethnicity information are reapportioned to a racial and ethnic group based on the distribution of HIV diagnoses, which do not have missing race and ethnicity data in NCHHSTP AtlasPlus.
Scenario 5	Reallocation based on information from syphilis diagnoses within a state. Within a state, diagnoses with missing race and ethnicity information are reapportioned to a racial and ethnic group based on the distribution of syphilis diagnoses, which do not have missing race and ethnicity data in NCHHSTP AtlasPlus.

^a^In 2018, the number of chlamydia and gonorrhea diagnoses stratified by race and ethnicity was not available for Connecticut, and this state was excluded from all scenarios. In addition, the number of HIV diagnoses by race and ethnicity is suppressed for New Hampshire in 2018, and the rates for New Hampshire were not adjusted under scenario 4.

## Results

### Descriptive Trends

**Figure 1:**
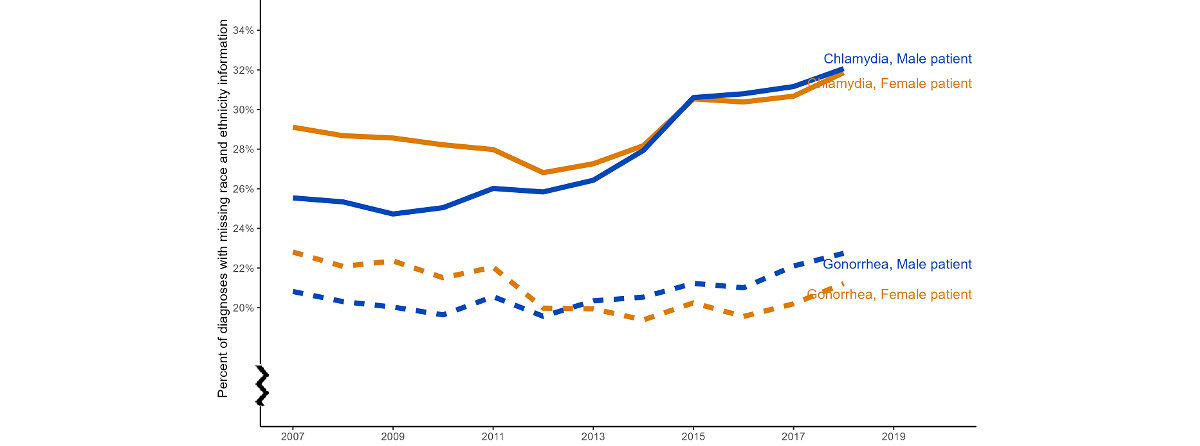
Percentage of reported chlamydia and gonorrhea diagnoses with missing race and ethnicity information in the United States (2007-2018). All 50 states and the District of Columbia are included. The national counts were developed by summing all counts from the states.

Figure 1 shows the annual trend of the percentage of missing race and ethnicity information among reported chlamydia and gonorrhea diagnoses by sex in 50 states and the District of Columbia from 2007 to 2018. The 2 solid lines represent the trends among male and female patients for reported chlamydia diagnoses, and the 2 dashed lines represent the trends among male and female patients for gonorrhea diagnoses. The percentage of missing race and ethnicity information was higher for chlamydia compared with gonorrhea. For each infection, female participants had a higher percentage of missing race and ethnicity data in 2007. The percentage of missing race and ethnicity information in reported gonorrhea diagnoses among female patients decreased over time (Cochran-Armitage 2-sided test for trend: *Z*=28.71; *P*<.001), but the corresponding indicator increased for reported gonorrhea diagnoses among male patients (*Z*=−29.21; *P*<.001). This resulted in a higher percentage of missing race and ethnicity information in reported gonorrhea diagnoses among male patients than among female patients in 2018. The percentage of missing race and ethnicity information in reported chlamydia diagnoses increased among both male patients (*Z*=−127.97; *P*<.001) and female patients (*Z*=−74.08; *P*<.001). However, the increasing trend was sharper for male patients, which resulted in closing the gap between the percentage of missing race and ethnicity information in reported chlamydia diagnoses among male and female patients in 2018.

Table 2 presents summary statistics of the percentage of missing race and ethnicity information among reported chlamydia and gonorrhea diagnoses for male and female patients in 50 states and the District of Columbia from 2007 to 2018. The results are stratified by female and male patients. For each year, the percentages reflect summary statistics of missingness across the 50 states and the District of Columbia. Overall, the reported chlamydia diagnoses had a higher frequency of missing race and ethnicity information than gonorrhea diagnoses, but differences in missingness between male and female patients were not remarkable. There was no clear trend when examining the median values of the percentage of missing racial and ethnic information across states. The range of missing data changed across states, with the minimum values remaining near 0% in all years for both infections but the maximum values increasing over time.

**Table 2:** Percentage of reported chlamydia and gonorrhea diagnoses with missing race and ethnicity information in 50 states and District of Columbia (2007-2018).

Sex and year		Chlamydia^a^		Gonorrhea^a^	
		Value, median (range; %)	IQR (Q1^b^-Q3^b^; %)	Value, median (range; %)	IQR (Q1^b^-Q3^b^; %)
**Female patients**					
	2007	23.6 (1.2-73.6)	(13.4-36.1)	18.5 (0-59.0)	(9.5-29.6)
	2008	25.5 (0-64.1)	(13.7-36.9)	22.6 (0-44.0)	(8.2-30.2)
	2009	24.3 (3.1-64.3)	(15.0-35.2)	19.6 (0-45.5)	(11.1-30.4)
	2010	29.2 (3.5-60.7)	(17.3-34.7)	21.0 (0-45.0)	(11.7-26.5)
	2011	27.5 (0.9-57.0)	(15.7-34.5)	19.7 (0-45.7)	(9.4-27.9)
	2012	23.5 (0-59.6)	(14.1-34.2)	15.9 (0-44.1)	(8.3-29.1)
	2013	22.8 (0.4-62.6)	(13.8-36.7)	16.5 (0-45.8)	(8.6-27.5)
	2014	27.4 (1.9-64.6)	(15.8-37.3)	18.2 (0-61.2)	(10.4-26.3)
	2015	29.2 (1.4-88.8)	(14.2-40.6)	17 (0-92.9)	(9.1-26.4)
	2016	27.2 (0.1-76.7)	(14.5-37.6)	17.8 (0-70.6)	(8.4-25.6)
	2017	26.5 (0.2-92.5)	(14.0-39.3)	17.4 (0.1-91.8)	(7.9-26.1)
	2018	26.9 (0.1-96.2)	(15.3-38.1)	16.9 (0.1-94.1)	(9.7-25.8)
**Male patients**					
	2007	19.9 (0.8-65.3)	(12.3-32.8)	17.4 (1.3-62.1)	(8.8-27.5)
	2008	20.7 (0-51.3)	(12.4-33.2)	22.4 (0-41.4)	(9.3-27.9)
	2009	21.4 (3.9-53.9)	(14.9-32.6)	18.2 (0-43.1)	(11.7-27.0)
	2010	24.3 (3.5-50.2)	(17.7-32.4)	21.2 (0-48.7)	(11.9-26.2)
	2011	23.9 (0.9-51.0)	(15.2-33.1)	18.2 (0-39.8)	(9.9-25.5)
	2012	23.5 (0-55.8)	(13.4-31.0)	16.1 (0-43.4)	(9.5-25.2)
	2013	24.6 (0.4-65.7)	(13.4-32.9)	18.6 (0-48.0)	(8.9-25.5)
	2014	25.2 (1.7-70.2)	(15.4-33.3)	20.1 (0-67.7)	(9.9-25.4)
	2015	26.8 (1.4-88.5)	(14.7-37.5)	17.6 (0.2-92.3)	(9.9-25.7)
	2016	27.3 (0.2-77.0)	(14.6-36.0)	18.2 (0.1-70.1)	(9.9-24.9)
	2017	25.9 (0.2-89.2)	(15.1-36.8)	17.4 (0.1-86.7)	(9.6-26.8)
	2018	29.5 (0.1-92.2)	(16.3-37.0)	17 (0-91.3)	(10.8-25.7)

^a^The observations are the percentage of diagnoses with missing racial and ethnic information in the 50 states and the District of Columbia.

^b^Q1 and Q3 are the first and third quartiles (the 25th and 75th percentiles, respectively).

### Scenario Analysis

**Figure 2:**
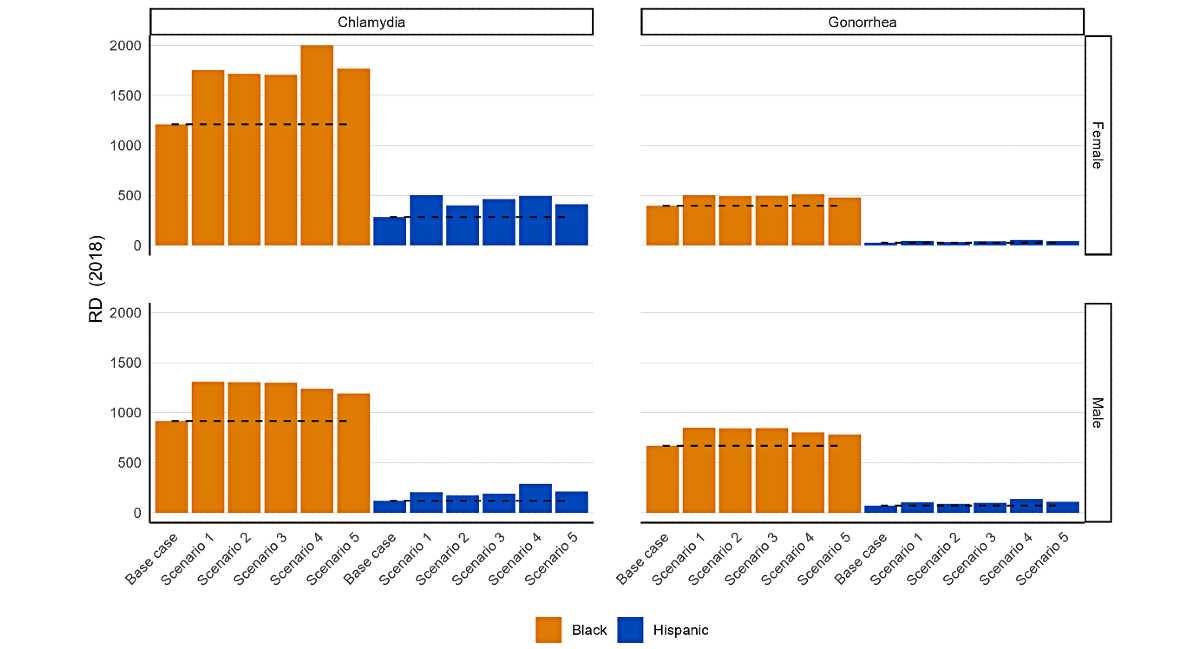
Estimated Black-White and Hispanic-White rate differences (RDs) for chlamydia and gonorrhea under 5 scenarios to impute race and ethnicity for reported diagnoses with incomplete demographic data (2018).

Figure 2 shows how the absolute Black-White and Hispanic-White disparity measures changed under each imputation scenario for the 2018 data, with the calculated disparity measures presented in Table 3. The numerators comprise all reported diagnoses regardless of the mode of transmission. National counts were developed by summing all counts from the states, except Connecticut, for which the number of chlamydia and gonorrhea diagnoses were not available by race and ethnicity in 2018. The denominator is the population of the United States aged ≥15 years in the 50 states and the District of Columbia, except Connecticut. The dashed line represents the value for the base case. The 4 charts display the RDs for chlamydia (left), gonorrhea (right), female patients (top), and male patients (bottom). The orange bars represent Black-White RDs, and the blue bars represent Hispanic-White RDs. There are 6 bars for each RD to represent the base case and the 5 imputation scenarios. In the base case, the Hispanic-White RDs for both chlamydia and gonorrhea are smaller than the Black-White RDs (chlamydia, RD: 284.1 per 100,000 for female patients and 119.4 per 100,000 for male patients; gonorrhea, RD: 27.5 per 100,000 for female patients and 71.8 per 100,000 for male patients). Under each imputation scenario, the RD disparity measure was higher compared with the base case. For chlamydia, the Black-White RD increased by up to 789.1 per 100,000 among female patients and up to 394.3 per 100,000 among male patients. The Hispanic-White RD increased by up to 210.1 per 100,000 among female patients and up to 168.2 per 100,000 among male patients. For gonorrhea, the Black-White RD increased by up to 114.2 per 100,000 among female patients and up to 182.2 per 100,000 among male patients. The Hispanic-White RD increased by up to 25.9 per 100,000 among female vs and up to 60.5 per 100,000 among male patients.

**Table 3:** Estimated Black-White and Hispanic-White rate differences (RDs) and rate ratios (RRs) for chlamydia and gonorrhea under 5 scenarios to impute race and ethnicity for reported diagnoses with incomplete demographic data (2018).

		Chlamydia						Gonorrhea				
		Female			Male			Female			Male	
		RD	RR	RD		RR	RD		RR	RD		RR
**Black-White**												
	Base case	1210.8	5.1	917.3		7.2	396.2		7.1	669.4		9.0
	Scenario 1	1750.9	5.2	1311.6		7.2	502.0		7.2	851.6		9.0
	Scenario 2	1715.6	5.0	1305.5		7.1	492.6		7.0	844.5		8.9
	Scenario 3	1705.1	5.0	1300.4		7.0	494.8		7.0	846.3		8.8
	Scenario 4	1999.9	6.3	1241.1		7.2	510.4		7.6	803.3		8.7
	Scenario 5	1766.5	5.2	1192.6		6.5	479.9		6.8	783.2		8.1
**Hispanic-White**												
	Base case	284.1	2.0	119.4		1.8	27.5		1.4	71.8		1.9
	Scenario 1	501.8	2.2	205.2		2.0	44.0		1.5	102.8		2.0
	Scenario 2	400.3	1.9	171.9		1.8	32.7		1.4	88.2		1.8
	Scenario 3	462.9	2.1	192.0		1.9	40.1		1.5	98.0		1.9
	Scenario 4	494.2	2.3	287.6		2.4	53.4		1.7	138.3		2.3
	Scenario 5	412.4	2.0	212.5		2.0	42.3		1.5	108.4		2.0

**Figure 3:**
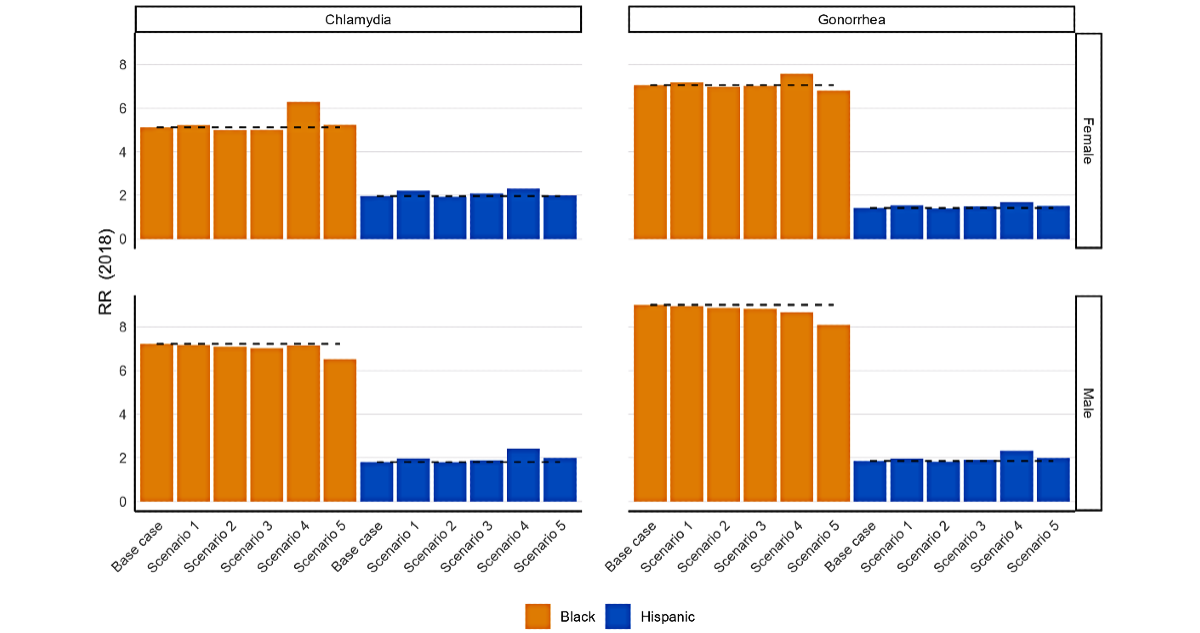
Estimated Black-White and Hispanic-White relative rate ratios (RRs) for chlamydia and gonorrhea under 5 scenarios to impute race and ethnicity for reported diagnoses with incomplete demographic data (2018).

Figure 3 displays the changes in the Black-White and Hispanic-White RRs under each scenario as a relative disparity measure, with the calculated disparity measures shown in Table 3. Its layout is similar to Figure 2, except that Figure 3 shows the RR outcome and a value of 1.0 would indicate there is no observed disparity. Without any adjustment for missing race and ethnicity information (base-case scenario), the Black-White RR for chlamydia in 2018 was 5.1 for female and 7.2 for male patients. The Black-White RR for gonorrhea was 7.1 for female and 9.0 for male patients. In the base case, the Hispanic-White RRs for both chlamydia and gonorrhea were smaller than the Black-White RRs (chlamydia, RR: 2.0 for female and 1.8 for male patients; gonorrhea: RR: 1.4 for female and 1.9 per for male patients). Under each imputation scenario, the RR remained stable compared with the base case. For chlamydia, the Black-White RR did not change remarkably in any scenario among the female or male patients. The Hispanic-White RR did not change remarkably in any scenario among female or male patients. Similarly, for gonorrhea, the Black-White RR did not change remarkably in any scenario among female or male patients. Moreover, the Hispanic-White RR showed no remarkable changes among female or male patients.

**Figure 4:**
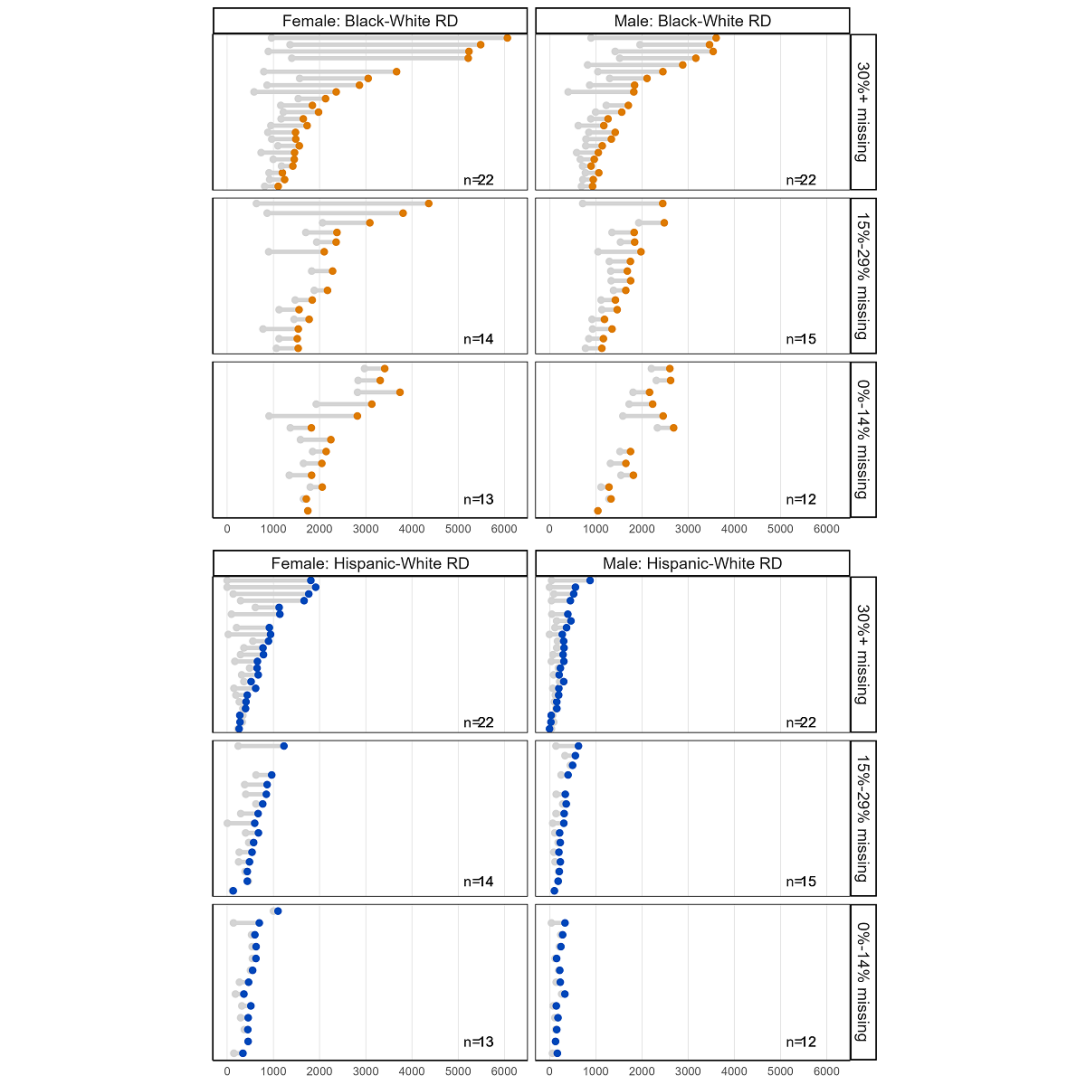
Illustration of changes in rate differences (RDs) as an absolute disparity measure for chlamydia across states with varying levels of missing race and ethnicity information using scenario 3 (reallocation based on information from states in the geographic region, for 2018).

At the state level, there was variation in how disparity measures changed under each scenario compared with the base case, with no adjustments for missing race and ethnicity information. Figure 4 presents dumbbell charts to illustrate how RDs for Black-White and Hispanic-White disparities among reported chlamydia diagnoses differ for each state under scenario 3 compared with the base case. This scenario and infection are presented for illustration, and all figures corresponding to other scenarios for each infection are available in the Multimedia Appendix 1. There is a dumbbell per state, excluding Connecticut and the District of Columbia. States were grouped into 3 categories based on their percentage of missing race and ethnicity information (0%-14% missing, 15%-29% missing, and ≥30% missing). The rate difference (x-axis) refers to the difference between the 2 diagnosis rates and is measured as diagnoses per 100,000 individuals. The gray dot is the base case, and the colored dot (orange or blue) is the scenario 3 value. The top and bottom panels display the RDs for the Black-White and Hispanic-White disparities, respectively. These patients were stratified according to sex. Each dumbbell represents the difference between the observed RD in the base-case scenario (gray dot) and the estimated RDs in scenario 3 (orange or blue dots). States that had larger discrepancies in their RDs after scenario 3 missing data adjustment had longer dumbbells. Under scenario 3, larger changes occurred in states with ≥30% of diagnoses with missing race and ethnicity information. The differences in RDs in scenario 3 versus the base case were more pronounced among female diagnoses and Black-White disparities. These qualitative conclusions were consistent when considering the other scenarios and gonorrhea (Multimedia Appendix 1).

**Figure 5:**
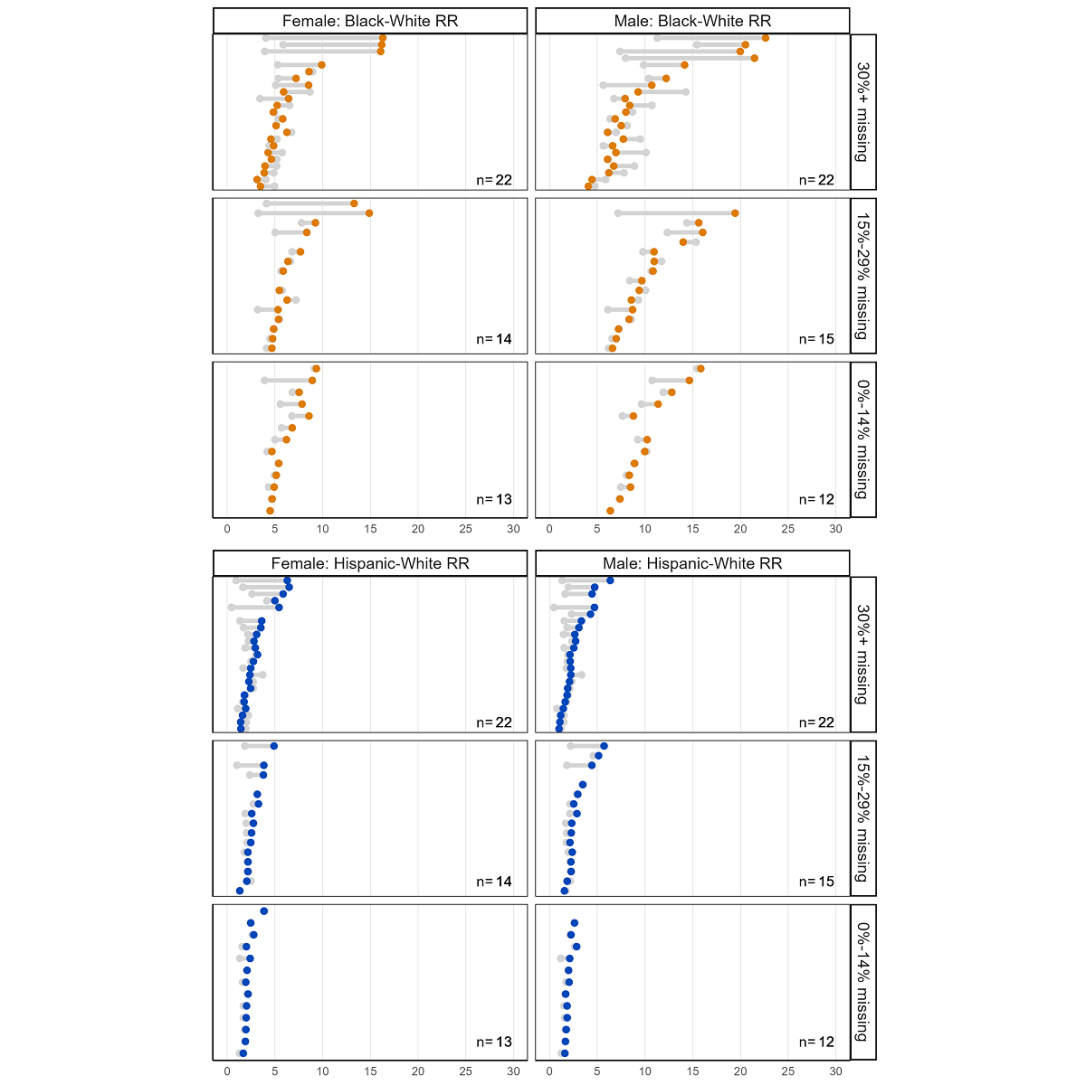
Illustration of changes in relative rate ratios (RRs) as relative disparity measures for chlamydia across states with varying levels of missing race and ethnicity information using scenario 3 (reallocation based on information from states in the geographic region, for 2018).

Figure 5 illustrates the changes in state-level RRs for chlamydia diagnoses, comparing scenario 3 with the base case. This is the same interpretation as that shown in Figure 4. Similar to the findings from Figure 4 (RDs), there were larger differences in RRs among states with a higher percentage of missing race and ethnicity information, and RR differences were more pronounced for Black-White disparities. However, there was no clear pattern based on sex. These qualitative conclusions were consistent when considering the other scenarios and gonorrhea (Multimedia Appendix 1).

## Discussion

### Principal Findings

To explore the impact of missing race and ethnicity information on disparity measures, we used 5 scenarios to redistribute diagnoses with missing race and ethnicity information and compared our estimated disparity measures to the base-case scenario that excluded diagnoses with missing demographic data. Nationally, the absolute disparity measures notably increased in the 5 imputation scenarios for both the infections and sexes. By contrast, at the national level, the relative disparity measures did not change notably under the 5 scenarios. States with higher percentages of missing race and ethnicity information experienced larger changes in their disparity measures when the information was imputed [26]. Our analysis provides several solutions to assess potential bias from missing demographic information. Choosing the best approach depends on the contextual factors of the affected population. For example, scenarios 4 and 5 may not be the best solutions for chlamydia and gonorrhea because of the differences between the race and ethnicity distributions of the chosen infections. However, these scenarios might be appropriate for other diseases that have similar race and ethnicity distributions. Similarly, scenarios 2 and 3 may not be appropriate for geographic regions that have a very different distribution of race and ethnicity than the population in their neighboring or regional states.

Prior research on cancer has shown how absolute and relative disparity measures can yield different conclusions about trends in population disparities and that the lack of a framework for measuring disparities can yield inconsistent communications about cancer-related health disparities and measuring progress toward national goals [15]. Absolute and relative measures take different perspectives on which aspects of population health to assess, and selecting a measure requires careful thinking about methodological issues as well as ethical and conceptual matters [15]. For example, a population health perspective prioritizes an absolute measure as a method to reflect the number of cases that would be averted from an intervention [15]. Our finding that the absolute measure was more sensitive to missing racial and ethnic information than the relative measure confirms that careful consideration is needed to select an appropriate disparity measure and interpret situations in which absolute and relative disparity measures diverge.

There are several reasons why the demographic data for reported chlamydia and gonorrhea diagnoses may be incomplete. Although standardized recommendations exist for collecting race and ethnicity information [27], demographic data collection is incomplete and inconsistent across jurisdictions and health care systems [28,29]. Incomplete collection of race and ethnicity information might result from individuals not disclosing information about their race and ethnicity because of mistrust or if they are provided with limited response options that do not match their self-identity [30]. Local health agencies’ efforts to follow up on reported diagnoses to collect additional demographic information can be costly and inefficient [31].

### Implications for Practice

In our experience and based on conversations with practitioners in the field, there are 3 primary sources of race and ethnicity information for newly diagnosed chlamydia and gonorrhea infections. First, diagnostic data may be obtained from laboratory reports that are automatically submitted to health departments, which frequently omit race and ethnicity information. Second, providers may submit case reports of notifiable conditions. Although these case reports should have race and ethnicity information, they may be incomplete, and passive surveillance systems based on laboratory data and case reports may have missing demographic information unless states can do active surveillance to obtain case reports on laboratory reports for which there is no matched case report. Third, race and ethnicity information may have been collected by disease intervention specialists through partner service interviews. However, interviews are less frequently conducted for gonorrhea and chlamydia following the CDC guidelines to prioritize HIV and syphilis for outreach [21]; furthermore, the high number of gonorrhea and chlamydia cases makes it infeasible to interview all individuals. Promising strategies for improving data quality include strengthening relationships with providers to improve the completeness of reporting, focusing on large-volume providers, and updating surveillance systems to use standardized electronic reporting.

Upstream and system-level solutions, such as enhanced electronic reporting, are needed to improve the availability of race and ethnicity information in public health surveillance systems, particularly when it is infeasible for public health workers to interview all cases. A past assessment of race and ethnicity information across different disease registries found that inconsistencies occurred more frequently among Hispanic populations and populations categorized as being in an “other” racial and ethnic group, suggesting that a more granular coding system for collecting demographic information might improve data completeness [32]. Furthermore, requiring race and ethnicity information in the initial data collection and simultaneously working with communities to improve surveillance instruments has been previously recommended to reduce the incompleteness of race and ethnicity information [30].

Informatics specialists can play important roles in designing cost-effective and interoperable solutions by defining standardized data elements, designing validation procedures, and automatically populating registries to enhance electronic reporting systems [5]. A recent case study showed that the automatic transfer of clinical data from an electronic health record system to public health surveillance improved the timeliness and quality of data with minimal manual intervention [26]. Moreover, collaboration with informatics specialists can improve the design and efficiency of data-entry systems for collecting more complete data. For example, systems can prevent progression until all required elements are filled out, and some aspects of the data entry can be automatically filled to avoid frustrating users with too many questions [3]. These types of informatics solutions could help enhance the electronic reporting of information required by public health agencies. Ultimately, obtaining more complete and accurate information on the front end is more efficient in terms of time and cost than assigning health department staff to locate persons with incomplete information for follow-up, particularly for high-morbidity diseases.

Our analysis highlights the importance of addressing missing data when calculating population rates and disparity measures. Although we focused on reported chlamydia and gonorrhea diagnoses among Black, Hispanic, and White populations, our findings likely apply to other outcomes or other population group comparisons. Missing data may lead to biased conclusions, especially if data are not missing at random across subpopulations [33]. When individual-level data are available, maximum likelihood and Bayesian multiple imputation methods are recommended to handle missing data [34]. For aggregate data, if spatial-level data are available, simple approaches, such as our 5 scenarios, can be used to impute missing data.

### Limitations

Our study has several limitations. First, there may be other approaches to impute the missing race and ethnicity information. Second, with our aggregate data, our analysis was not designed to assess the best imputation scenario but to illustrate the potential impacts of missing race and ethnicity information on health disparity measures. Finding the best imputation scenario is an important area for future research using individual-level data from medical records, claims data, or other sources matched with surveillance data. Third, we examined a limited number of racial and ethnic disparities because the number of reported chlamydia and gonorrhea diagnoses was small in the population groups other than those recorded as Black, Hispanic, or White, making it difficult to calculate stable estimates.

### Conclusions

Our analysis showed that the observed disparities are likely underestimated because of missing race and ethnicity information, particularly when using an absolute disparity measure. More complete race and ethnicity information is important to better understand the contributing causes of inequities and to monitor progress toward policy initiatives to reduce disparities. Addressing the missing demographic information in surveillance systems requires system-level solutions. However, as a short-term solution, local public health agencies can adapt imputation scenarios to adjust surveillance data for use in population indicators of health equity. Imputation scenarios can be integrated with the existing public health informatics infrastructure. Using these scenarios requires data analytics staff with knowledge of statistical analysis software, and there may be a limited ability to prioritize human resources for scenario analysis, particularly in local health departments or during public health emergencies such as COVID-19 or monkeypox. However, they do not require additional data or changes to the system design, making them useful short-term solutions for situations in which human resources are available.

## Appendix

Multimedia Appendix 1 Technical appendix with complete results for all scenarios and infections.

## Abbreviations

CDC: Centers for Disease Control and Prevention
RD: rate difference
RR: rate ratio
STI: sexually transmitted infection
