# Supplementary material for: Modeling the Potential Impact of Missing Race and Ethnicity Data in Infectious Disease Surveillance Systems on Disparity Measures: Scenario Analysis of Different Imputation Strategies
Source: JMIR Public Health Surveill. 2022 Nov 9;8(11):e38037. doi: 10.2196/38037 (PMC9685511; doi:10.2196/38037)
Supplement: Multimedia Appendix 1 [file publichealth_v8i11e38037_app1.docx]

**Modeling the Impact of Missing Race and Ethnicity Data in Infectious Disease Surveillance Systems on Disparity Measures: Scenario Analysis of Different Imputation Strategies**

**TECHNICAL APPENDIX**

This is the complete set of charts to show how the rate ratios and rate differences would change under the five scenarios compared to the base case for the two infections in 2018. There are 20 sets of charts, for each combination of the five scenarios, two outcomes (RR and RD), and two infections. Appendix Figures 1 through 10 display chlamydia disparity measures, and Appendix Figures 11 through 20 display gonorrhea disparity measures.

Within each figure, the top panel shows the comparison between the scenario and the base case for Black-White disparities (orange) and the bottom panel shows the comparison between the scenario and the base case for Hispanic-White disparities (blue). In each chart, there is one dumbbell per state, excluding Connecticut and District of Columbia. States are grouped based on what percentage of the chlamydia diagnoses had missing race/ethnicity information. For the rate ratio charts, the X-axis units are dimensionless (ratios) and for the rate difference charts, the X-axis scale is diagnoses per 100,000.

**Appendix Figure 1. Changes in rate differences for chlamydia under the Scenario 1 compared to the base case**

| 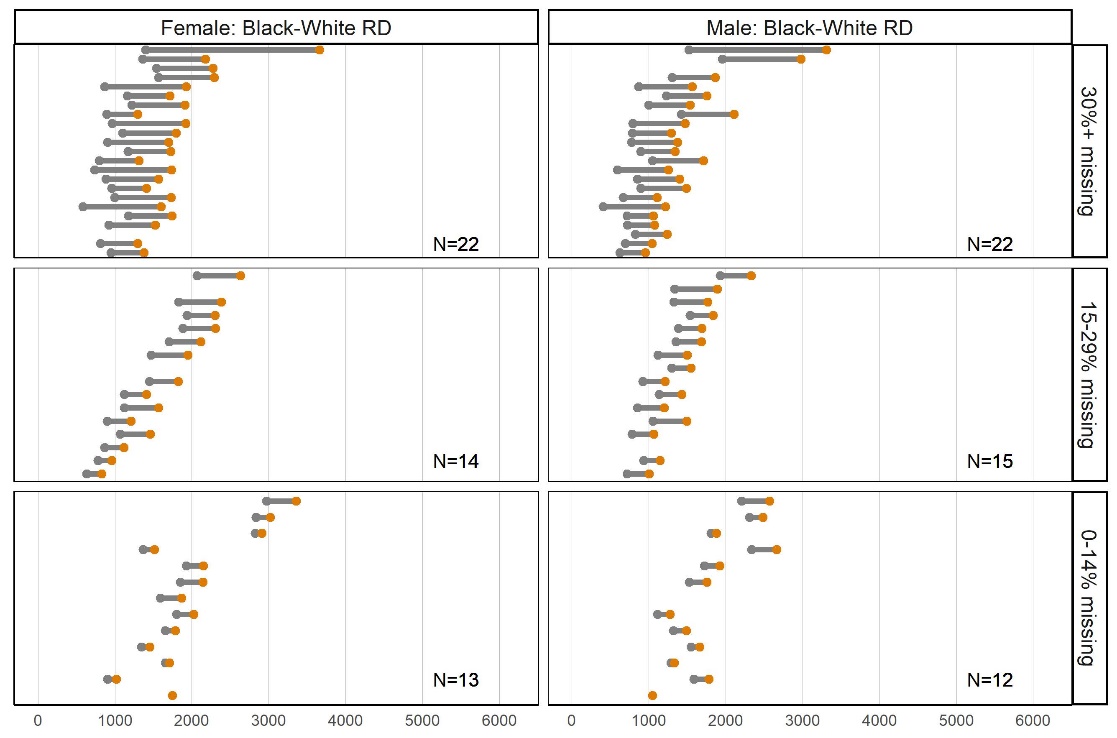 |
| --- |
| 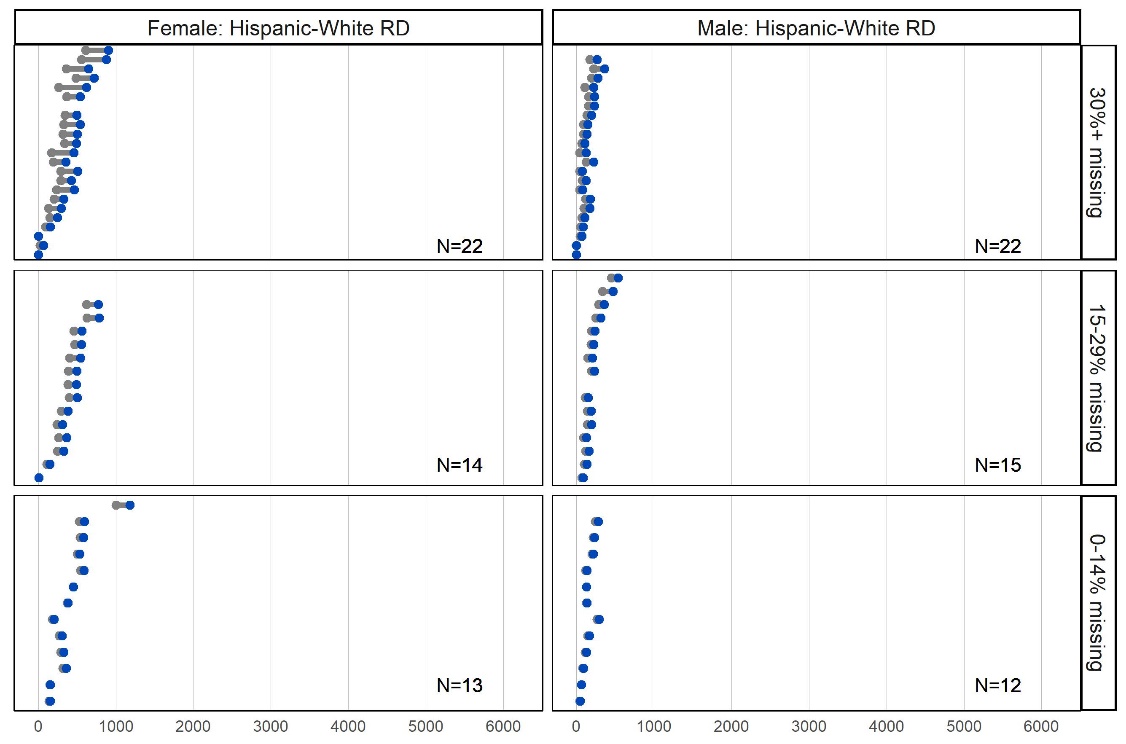 |

**Appendix Figure 2. Changes in relative rate ratios for chlamydia under the Scenario 1 compared to the base case**

| 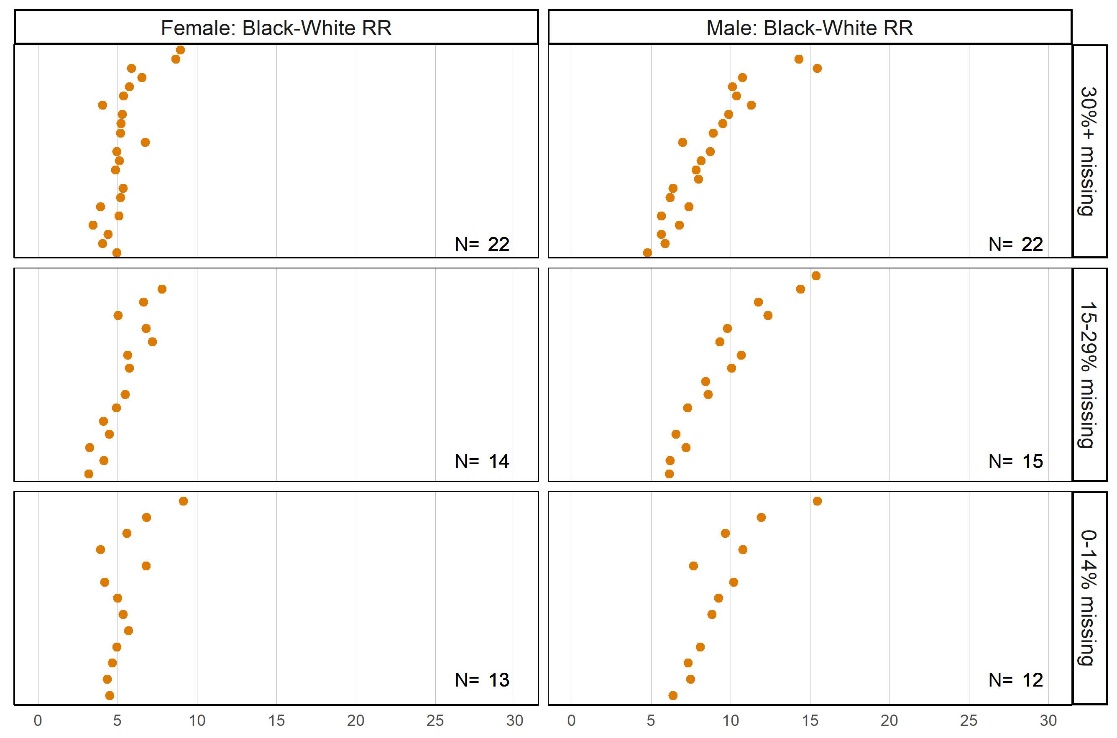 |
| --- |
| 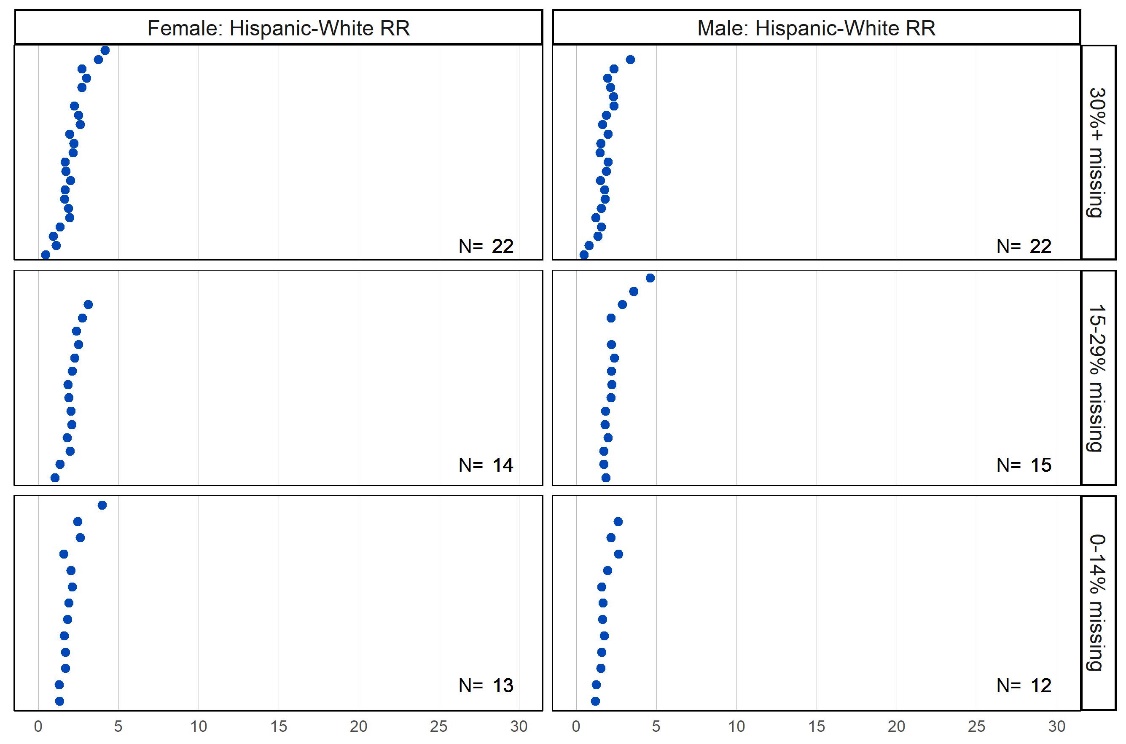 |

Notes: Rate ratios calculated for the Scenario 1 are not different from the base case because under the Scenario 1, we assumed that the distribution of diagnoses with missing race/ethnicity was the same as the distribution of diagnoses with known race/ethnicity information in the same state.

**Appendix Figure 3. Changes in rate differences for chlamydia under the Scenario 2 compared to the base case**

| 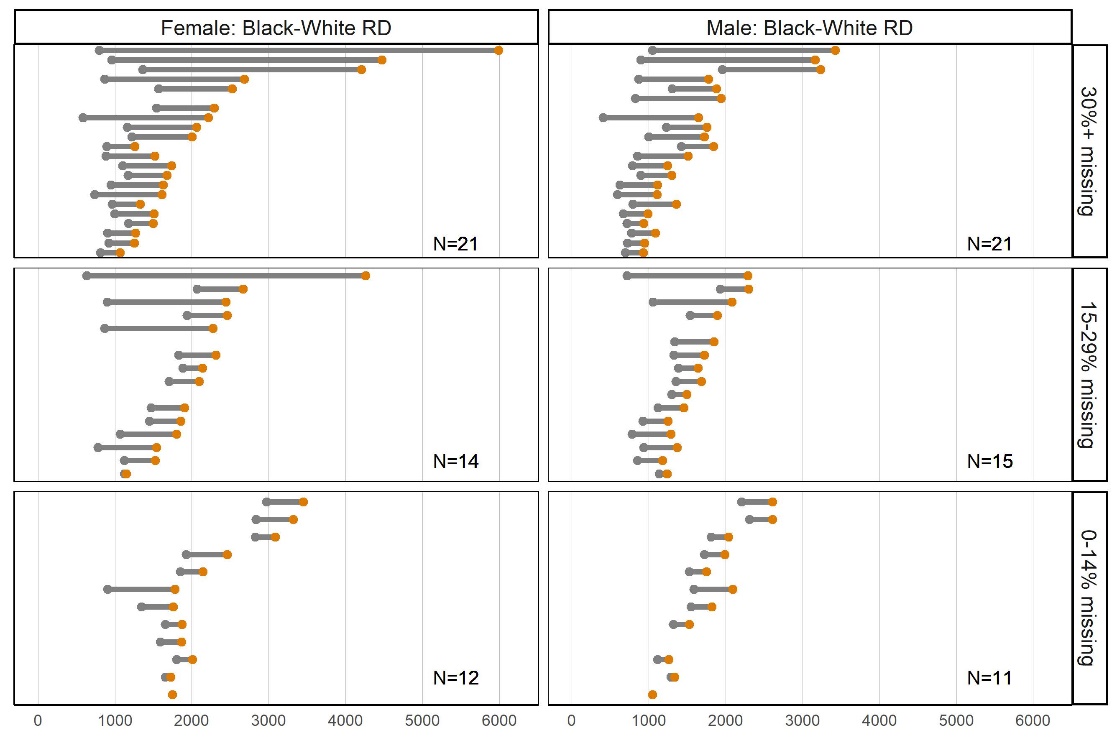 |
| --- |
| 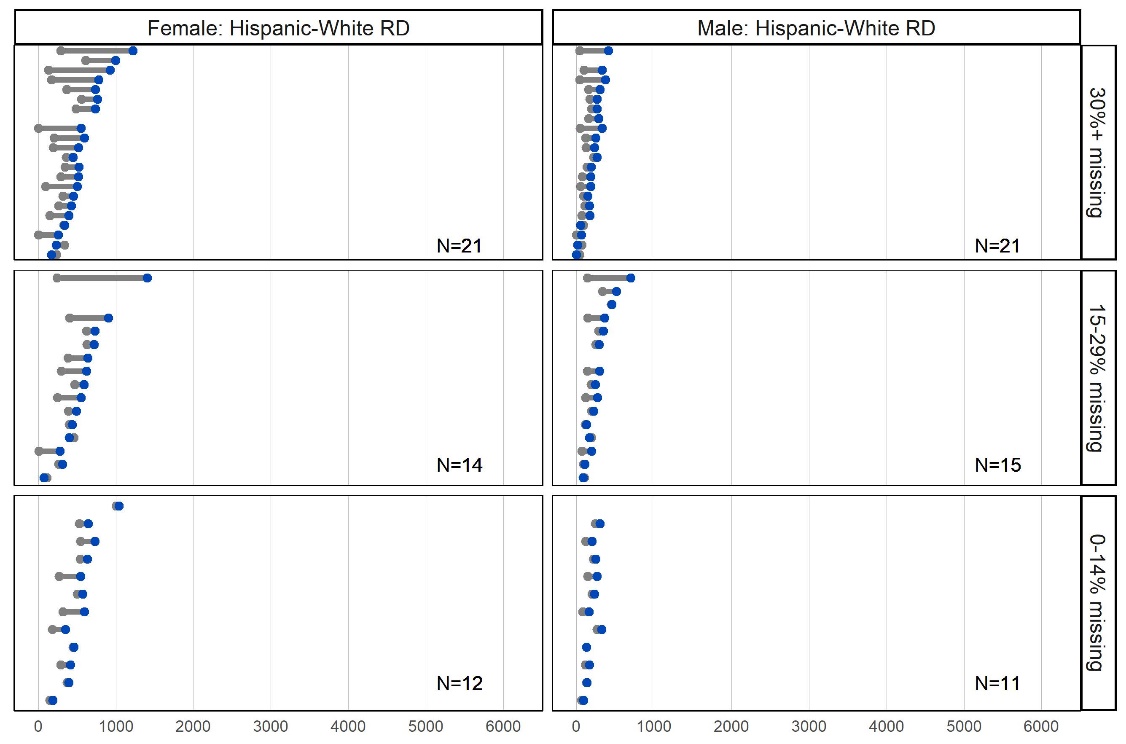 |

**Appendix Figure 4. Changes in relative rate ratios for chlamydia under the Scenario 2 compared to the base case**

| 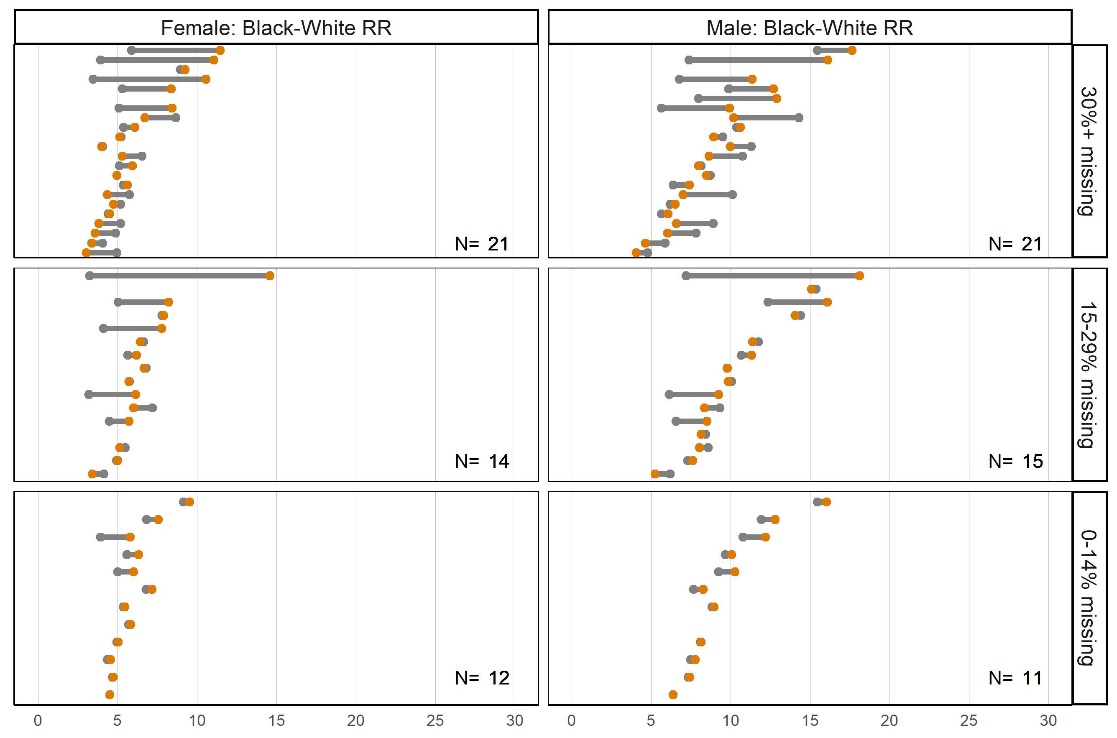 |
| --- |
| 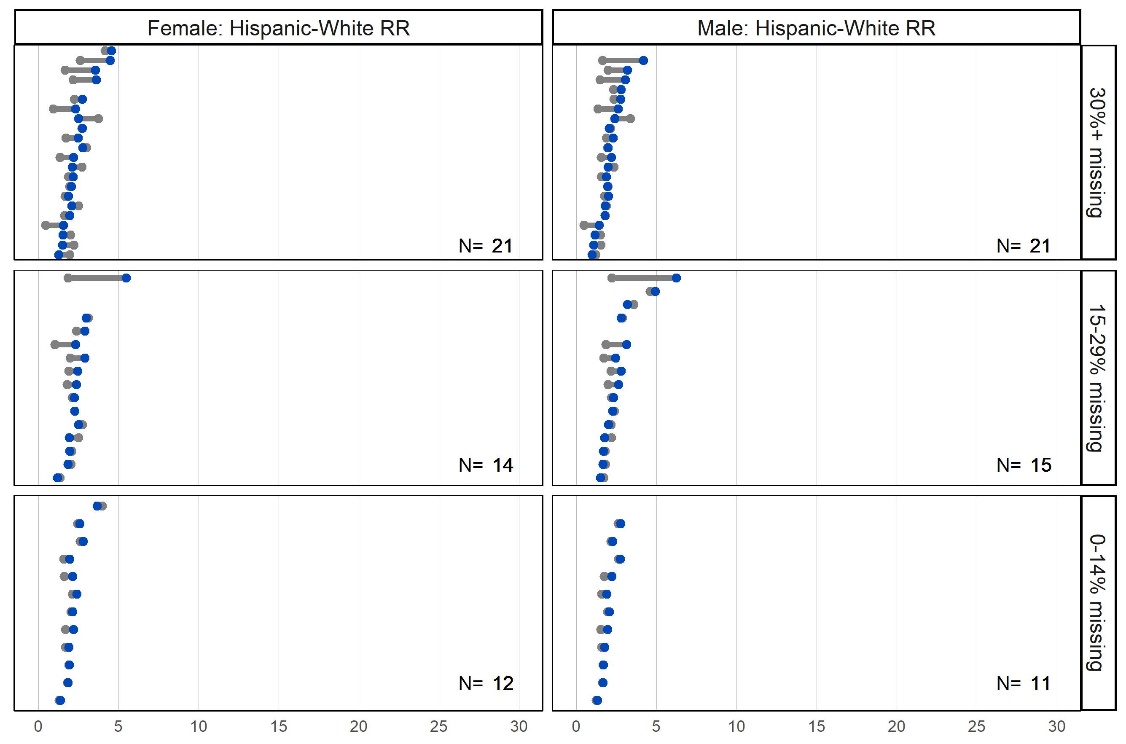 |

**Appendix Figure 5. Changes in rate differences for chlamydia under the Scenario 3 compared to the base case**

| 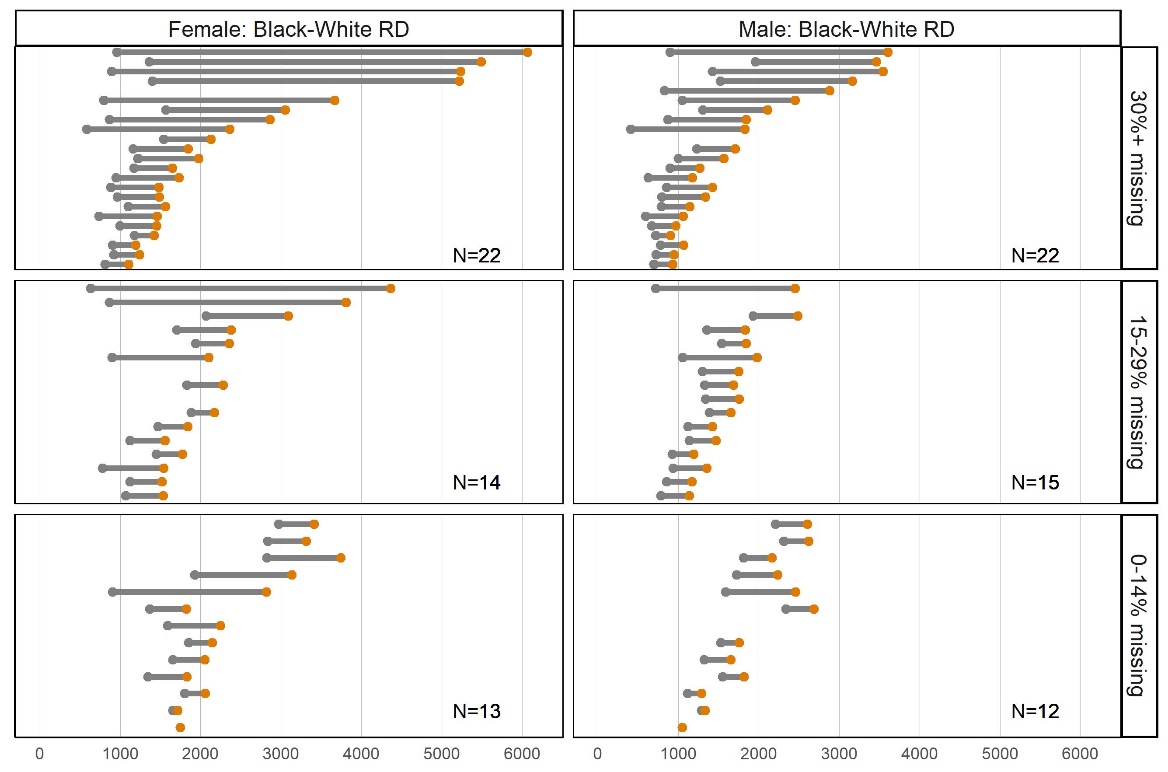 |
| --- |
| 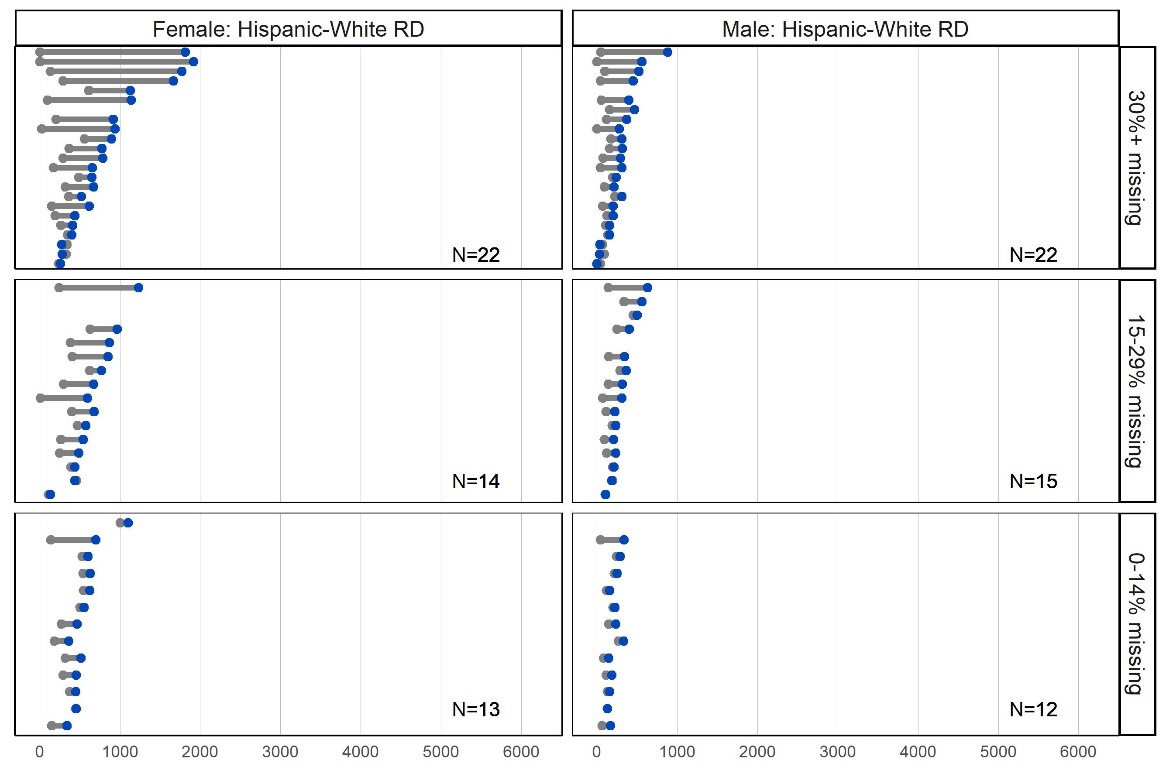 |

**Appendix Figure 6. Changes in relative rate ratios for chlamydia under the Scenario 3 compared to the base case**

| 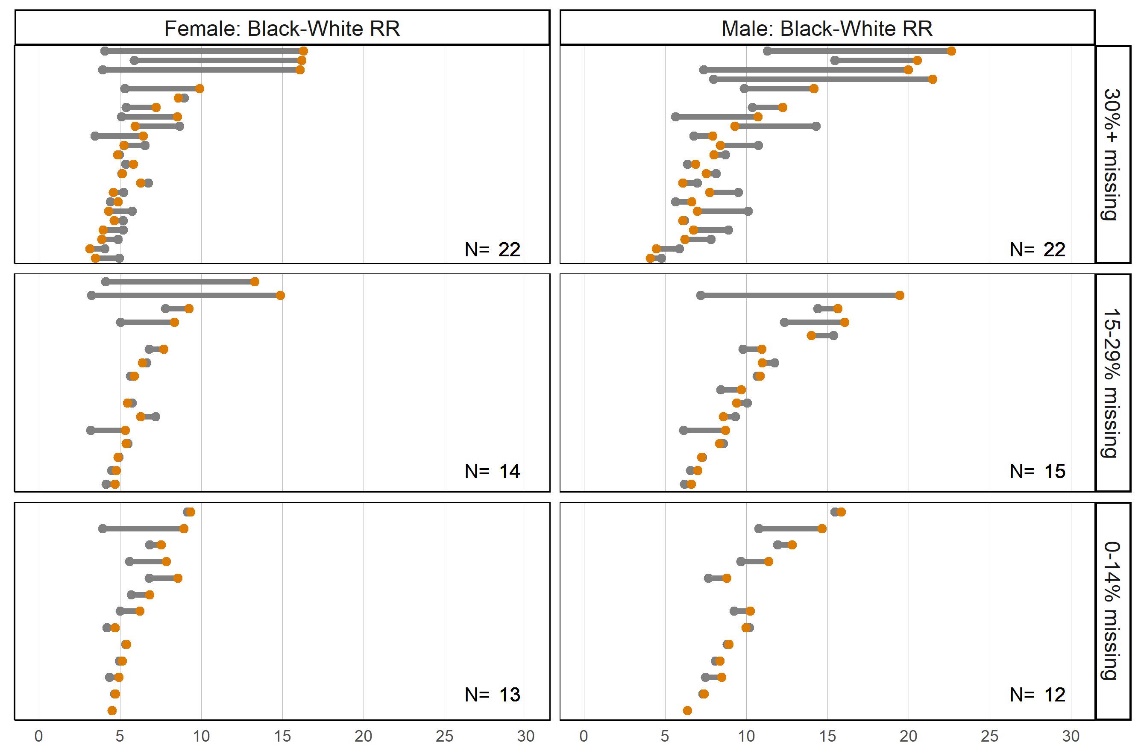 |
| --- |
| 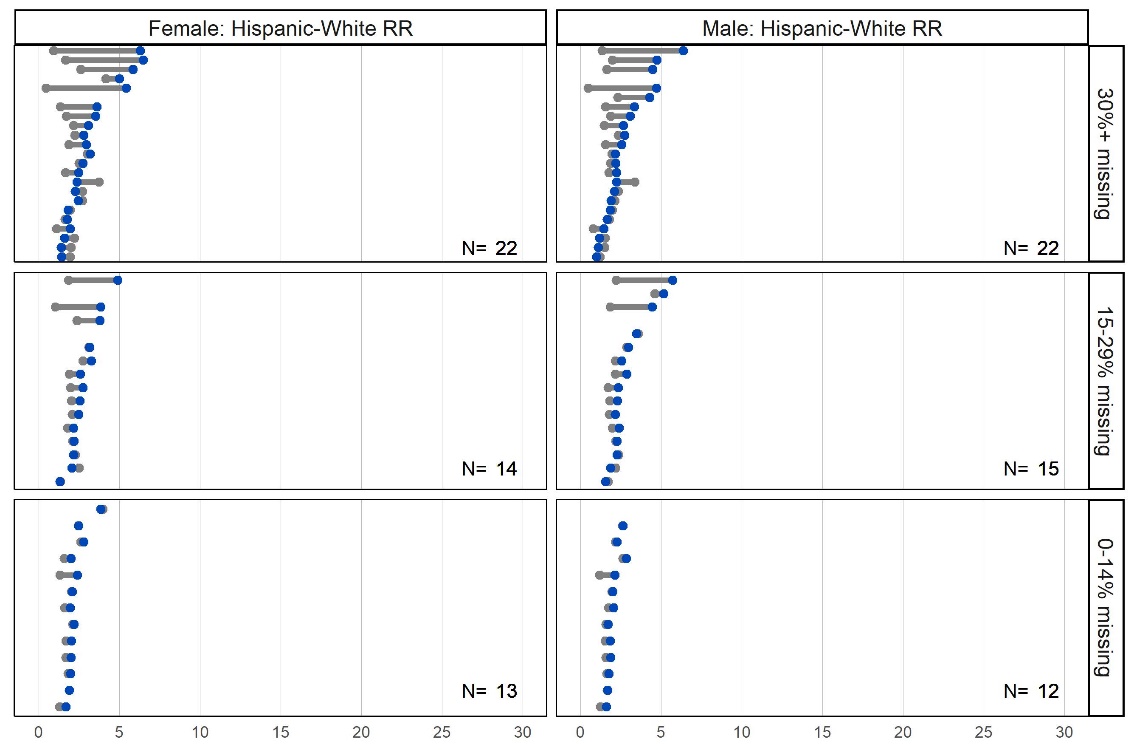 |

**Appendix Figure 7. Changes in rate differences for chlamydia under the Scenario 4 compared to the base case**

| 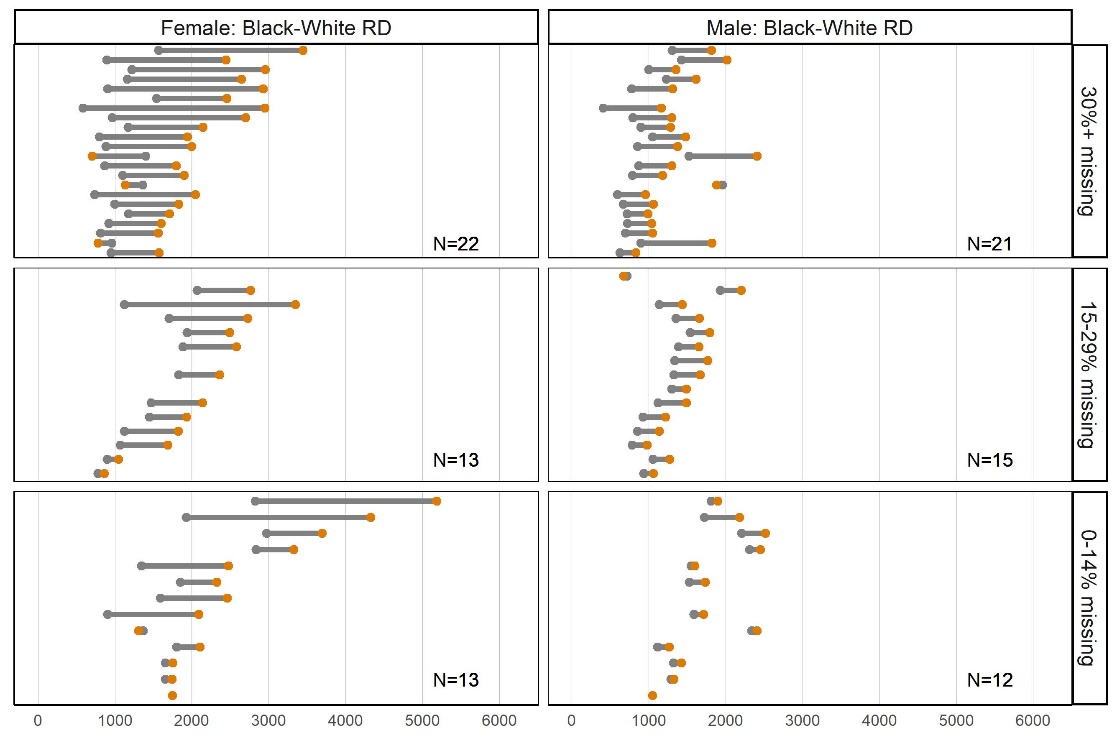 |
| --- |
| 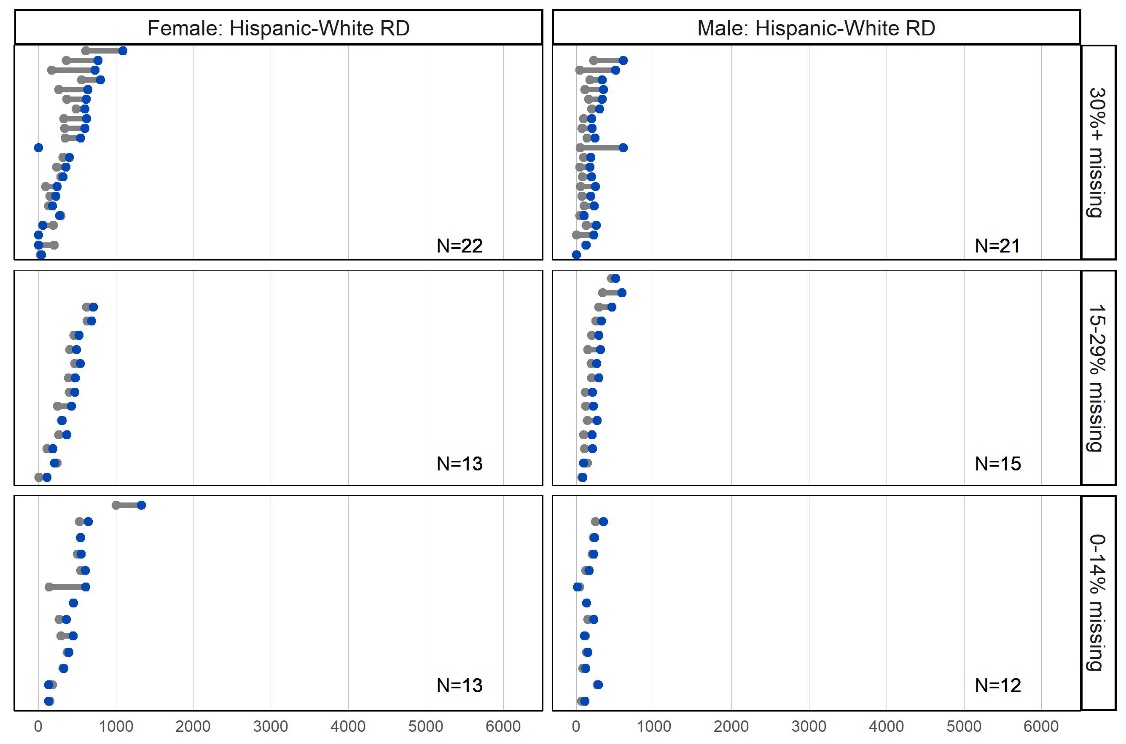 |

**Appendix Figure 8. Changes in relative rate ratios for chlamydia under the Scenario 4 compared to the base case**

| 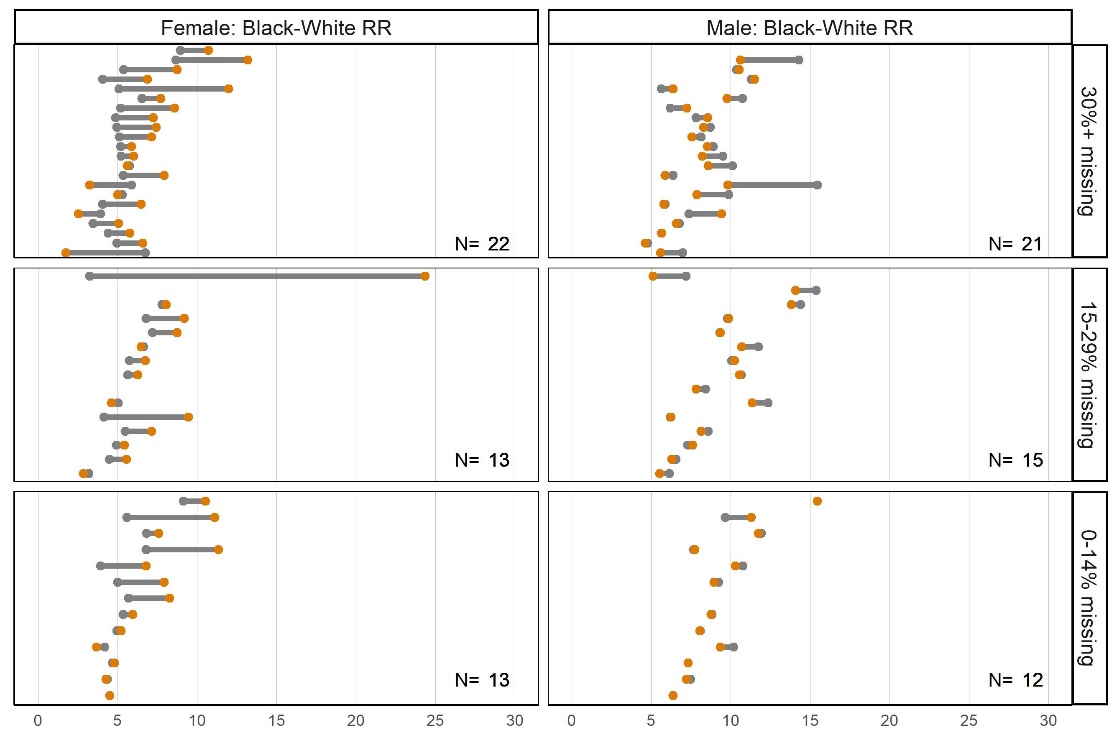 |
| --- |
| 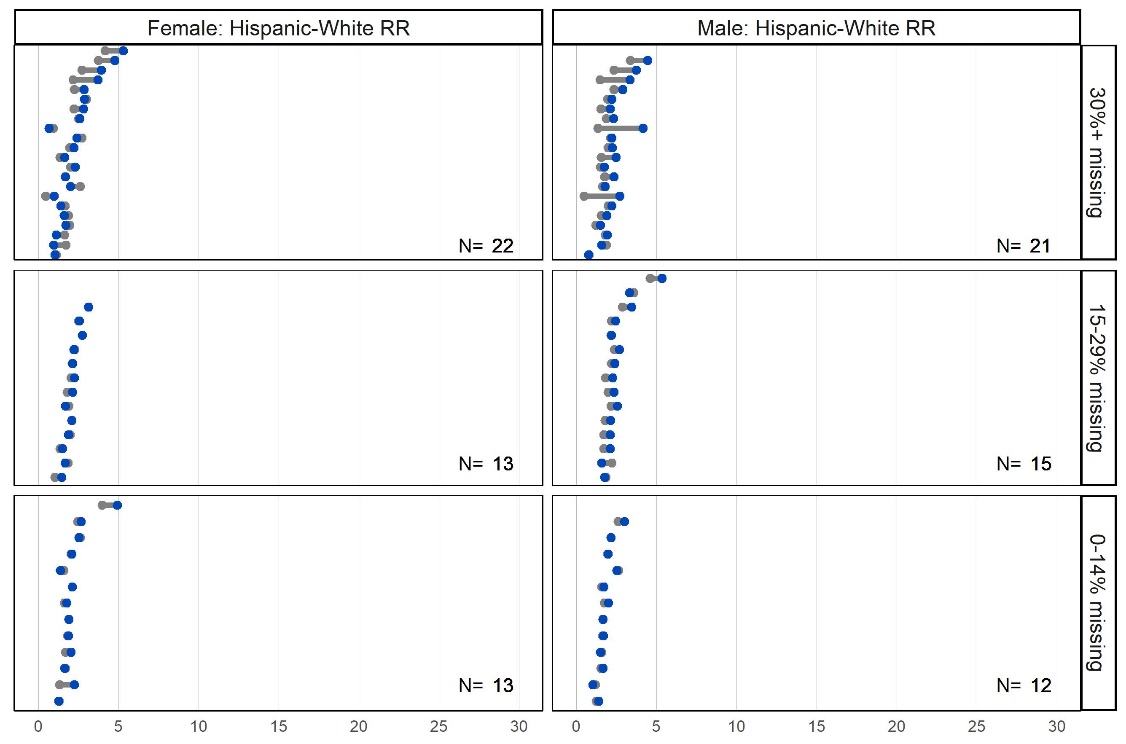 |

**Appendix Figure 9. Changes in rate differences for chlamydia under the Scenario 5 compared to the base case**

| 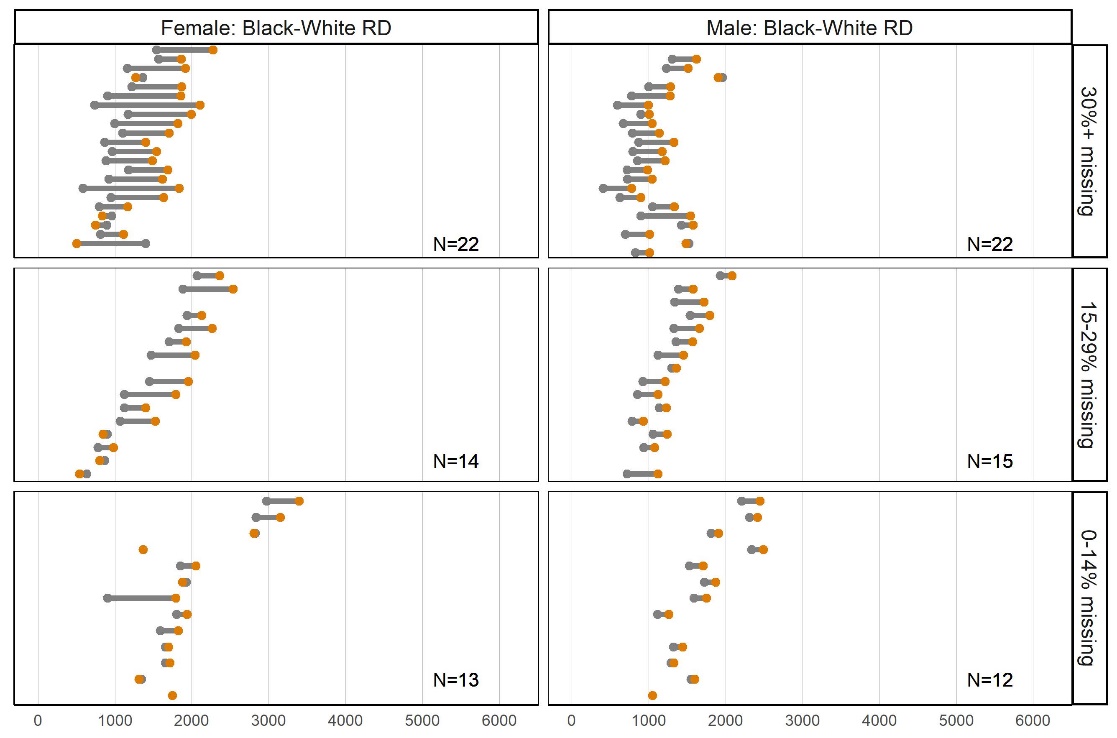 |
| --- |
| 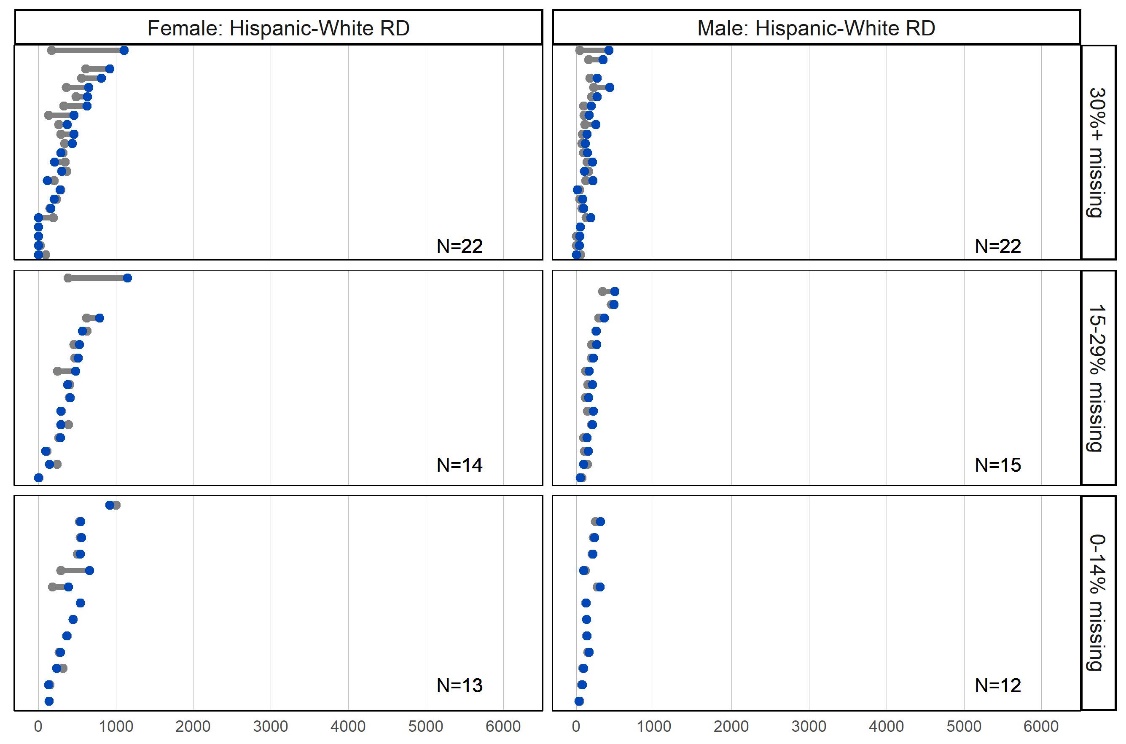 |

**Appendix Figure 10. Changes in relative rate ratios for chlamydia under the Scenario 5 compared to the base case**

| 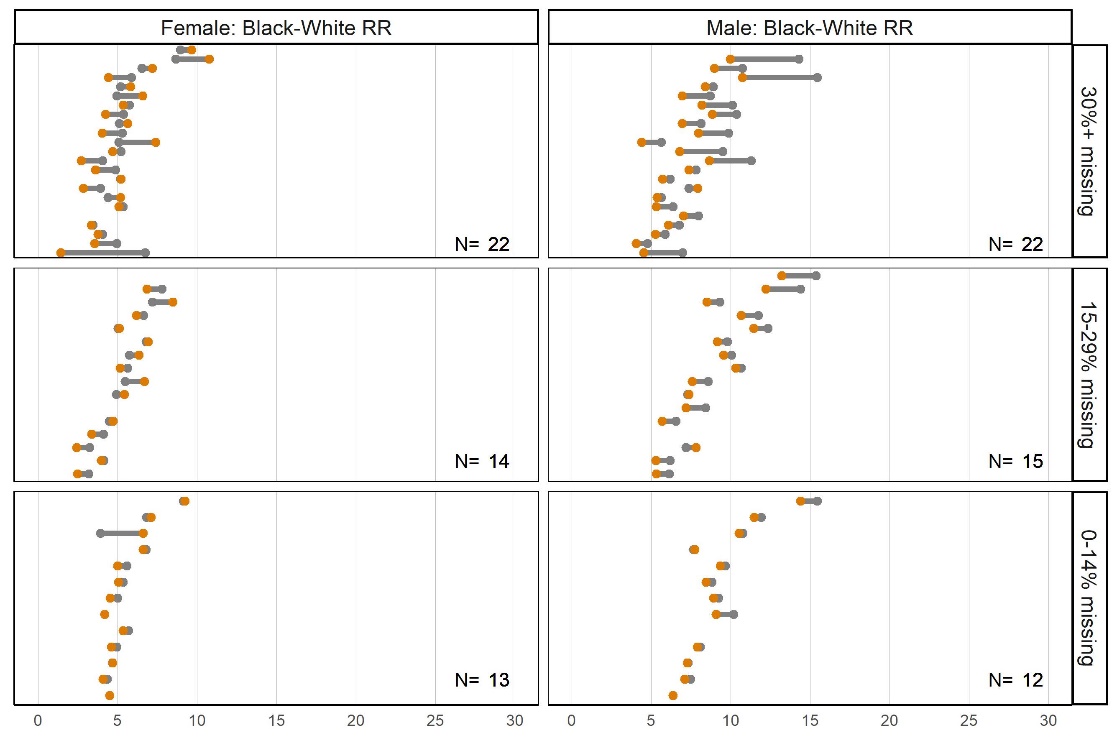 |
| --- |
| 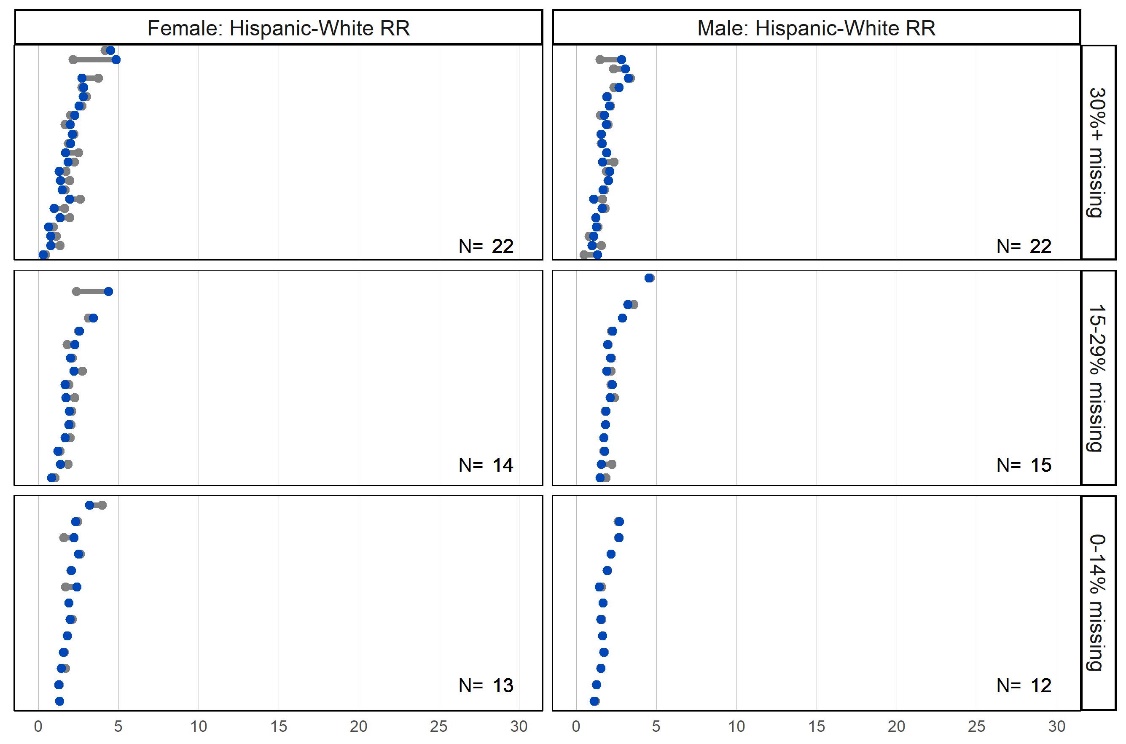 |

**Appendix Figure 11. Changes in rate differences for gonorrhea under the Scenario 1 compared to the base case**

| 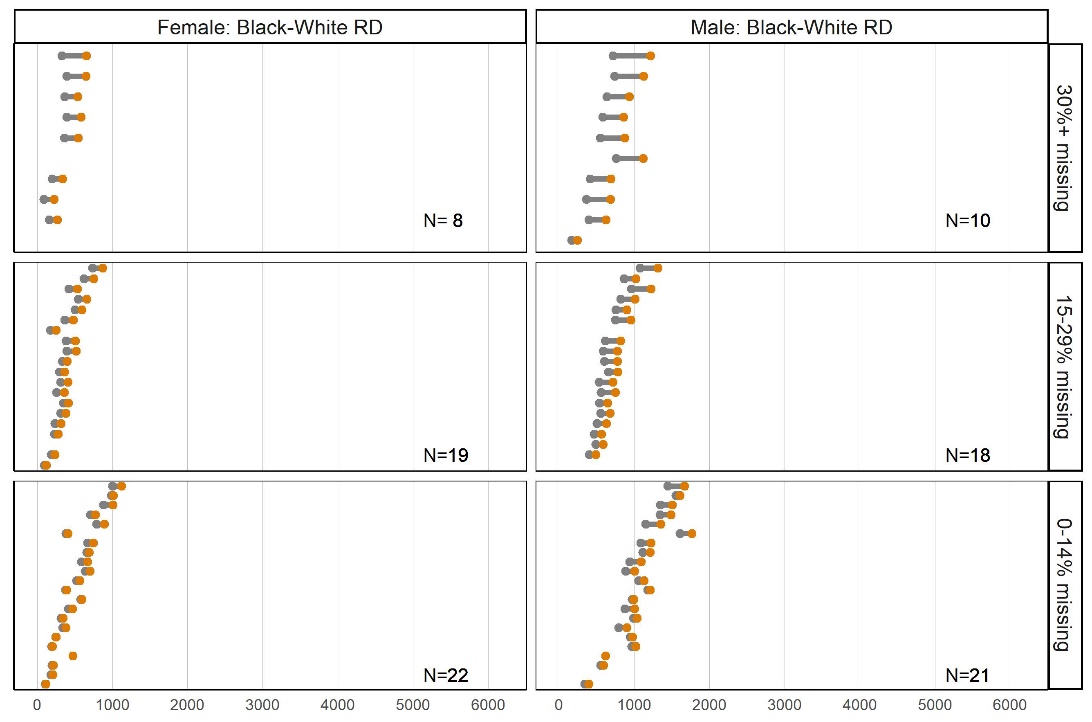 |
| --- |
| 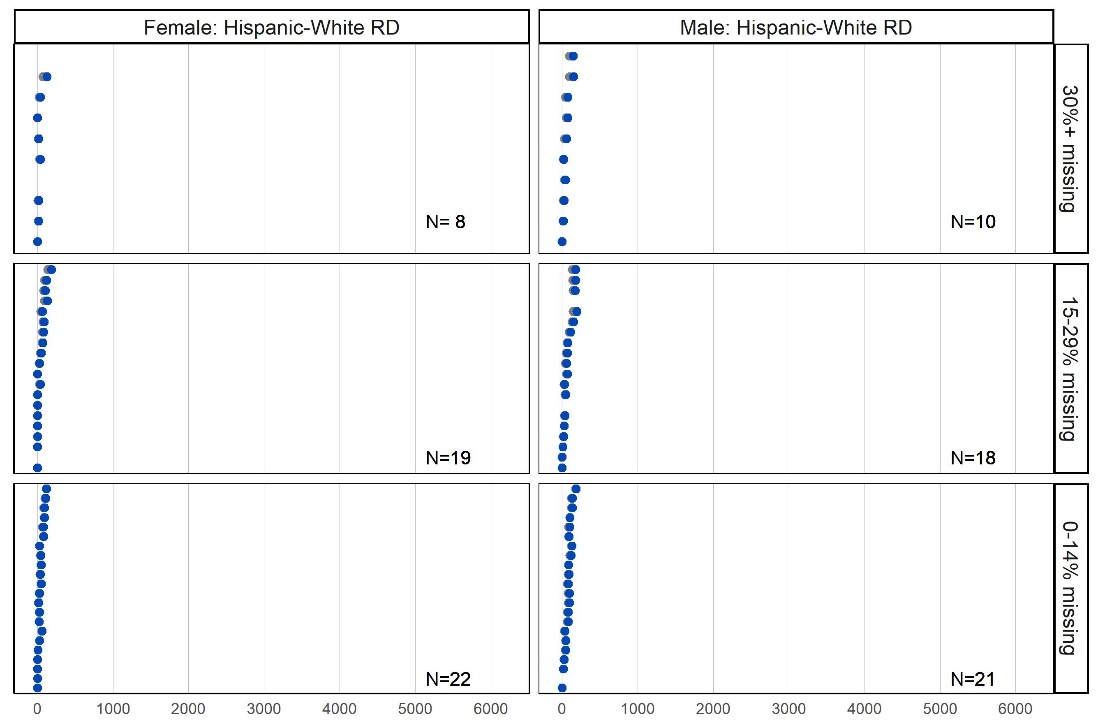 |

**Appendix Figure 12. Changes in relative rate ratios for gonorrhea under the Scenario 1 compared to the base case**

| 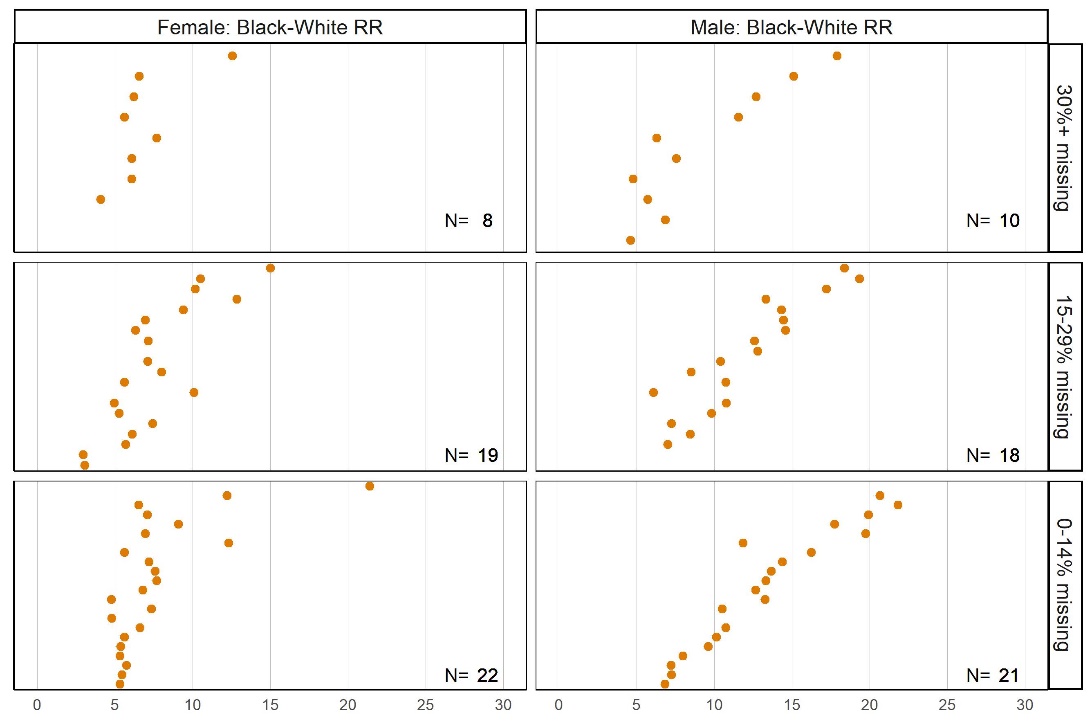 |
| --- |
| 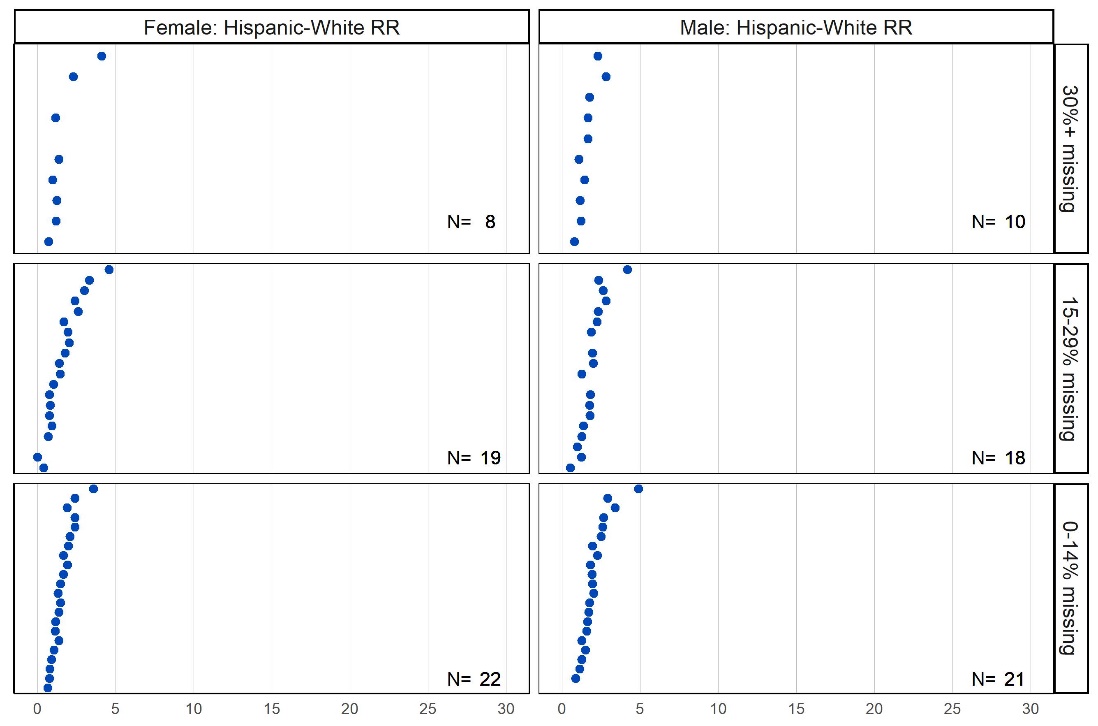 |

Notes: Rate ratios calculated for the Scenario 1 are not different from the base case because under the Scenario 1, we assumed that the distribution of diagnoses with missing race/ethnicity was the same as the distribution of diagnoses with known race/ethnicity information in the same state.

**Appendix Figure 13. Changes in rate differences for gonorrhea under the Scenario 2 compared to the base case**

| 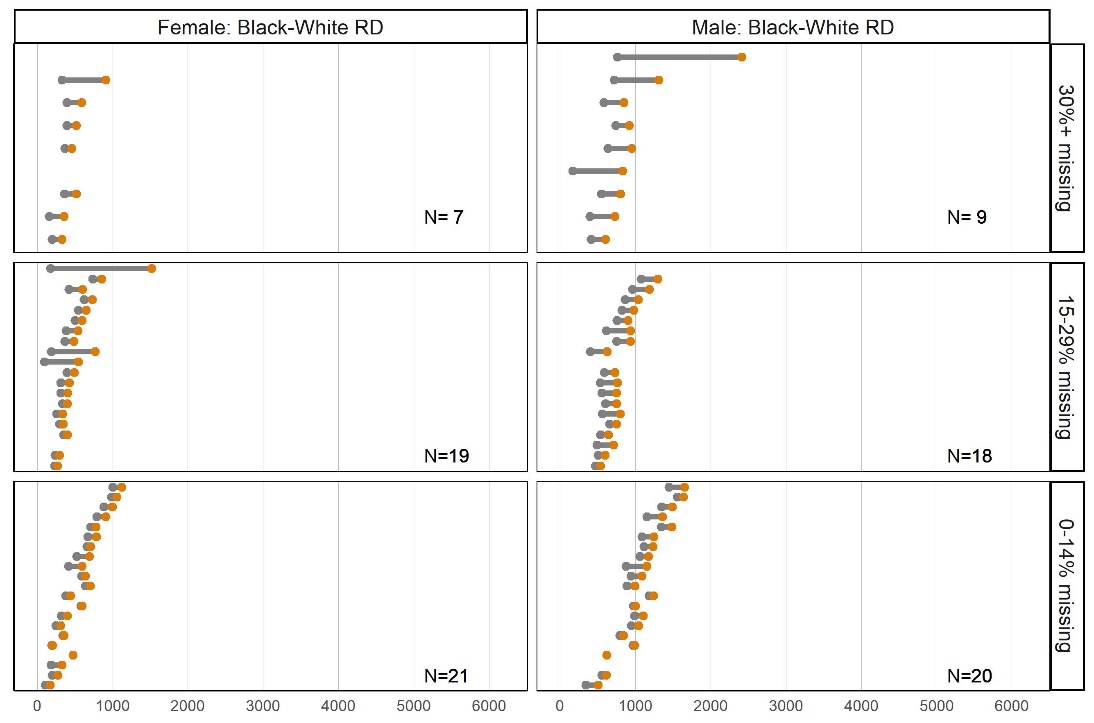 |
| --- |
| 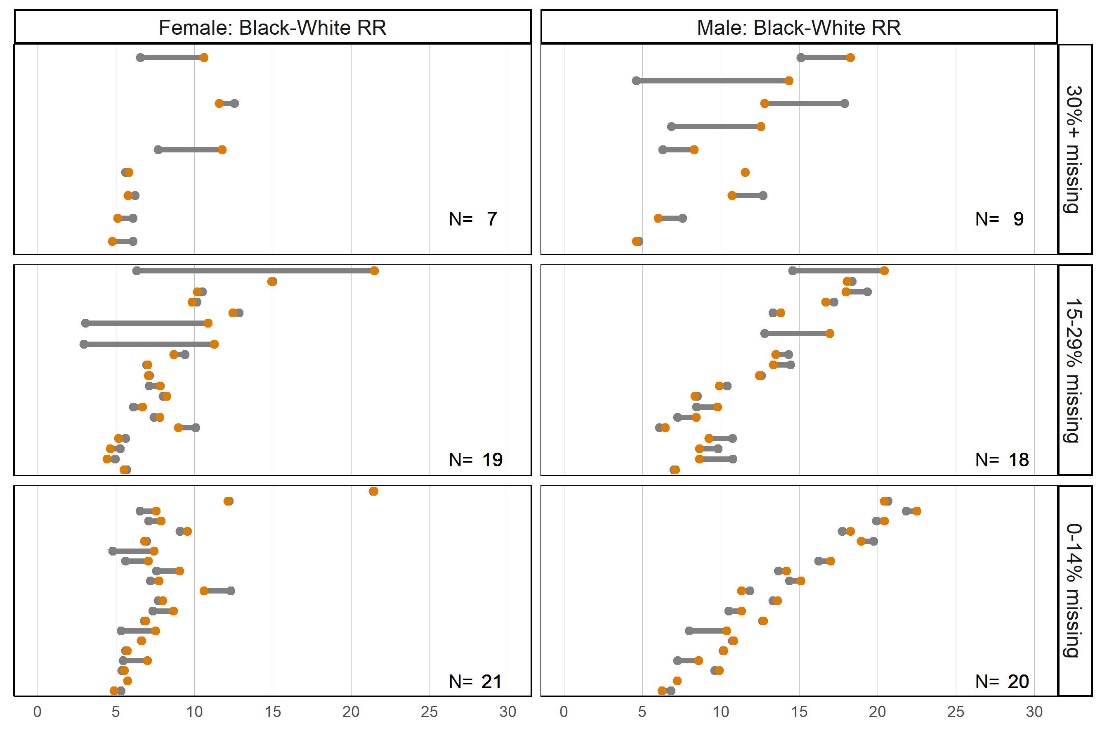 |

**Appendix Figure 14. Changes in relative rate ratios for gonorrhea under the Scenario 2 compared to the base case**

| 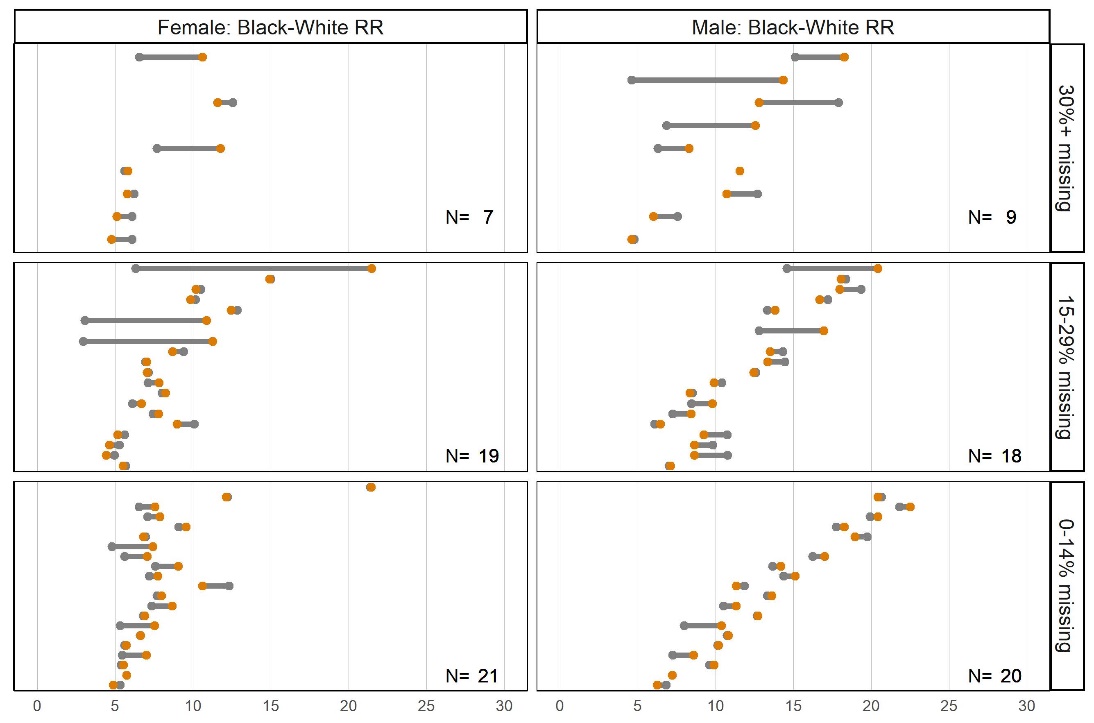 |
| --- |
| 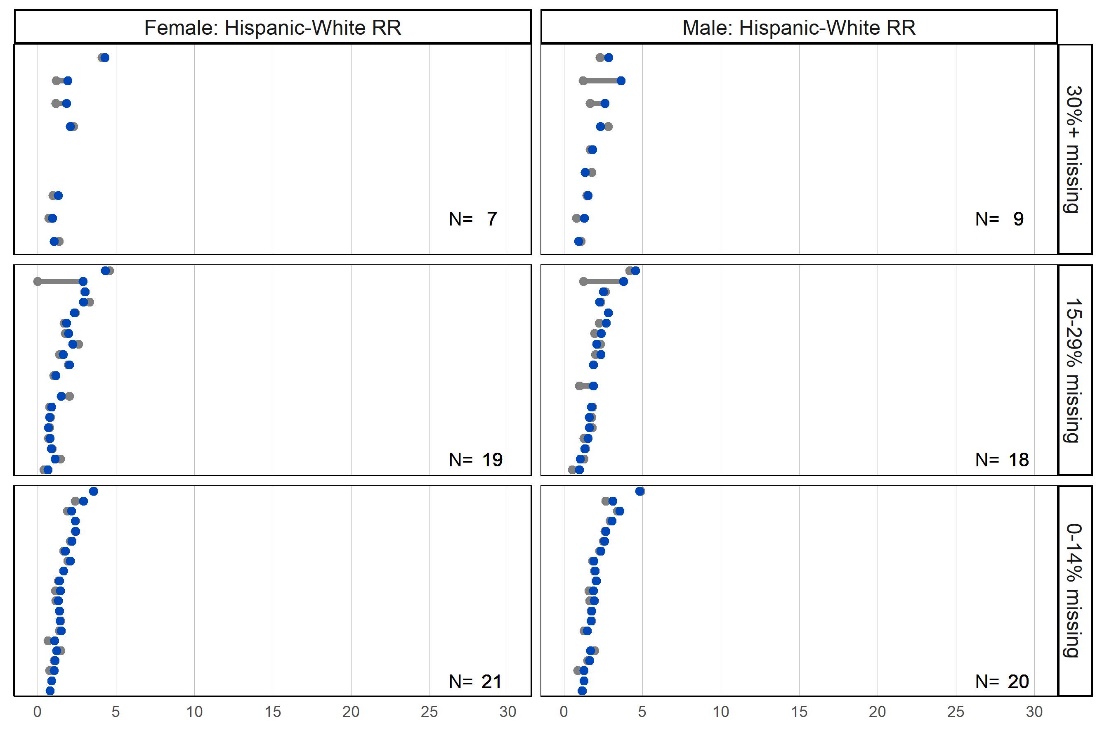 |

**Appendix Figure 15. Changes in rate differences for gonorrhea under the Scenario 3 compared to the base case**

| 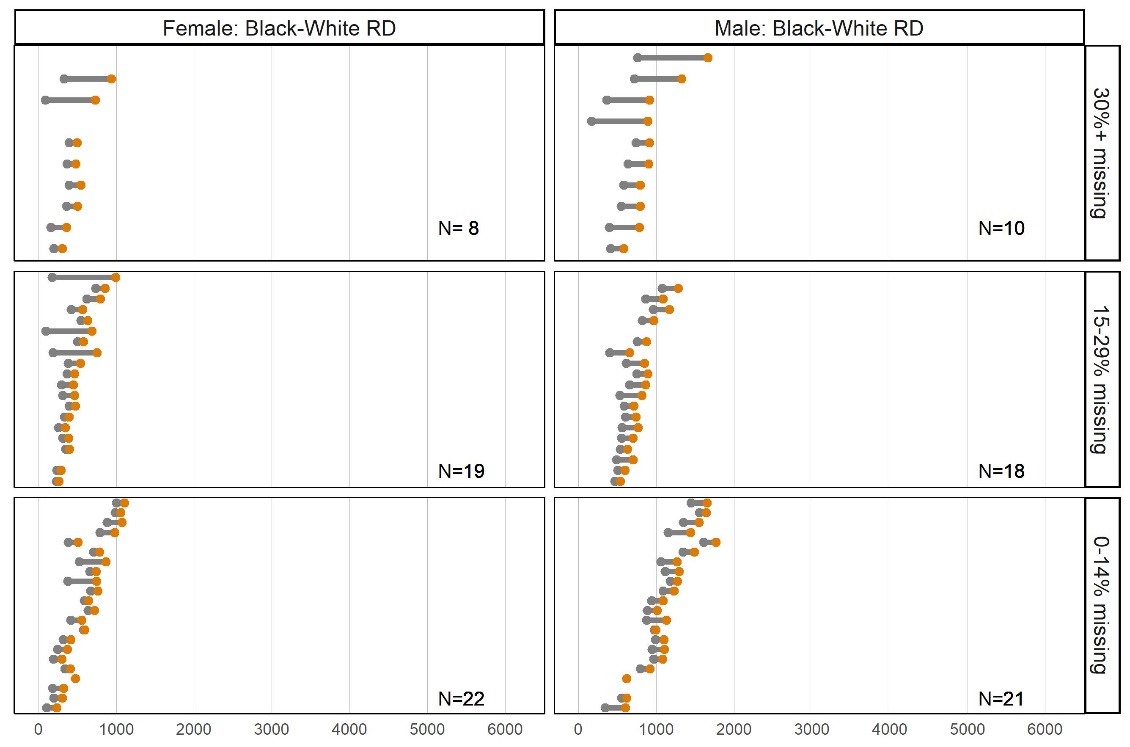 |
| --- |
| 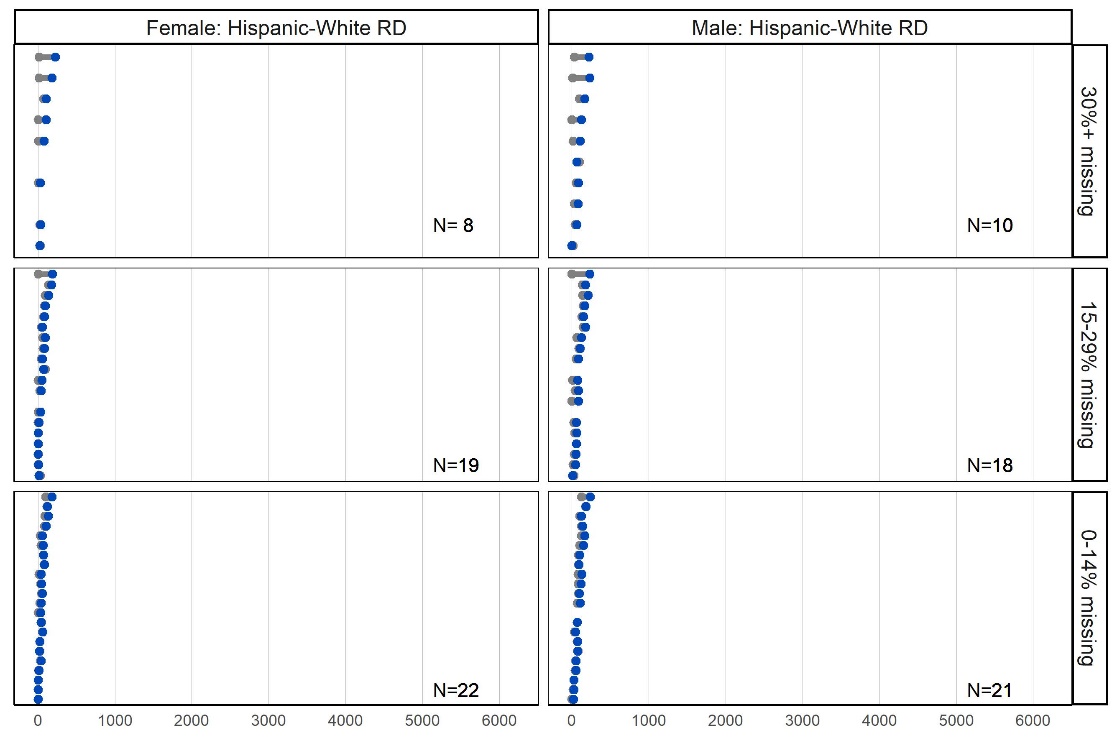 |

**Appendix Figure 16. Changes in relative rate ratios for gonorrhea under the Scenario 3 compared to the base case**

| 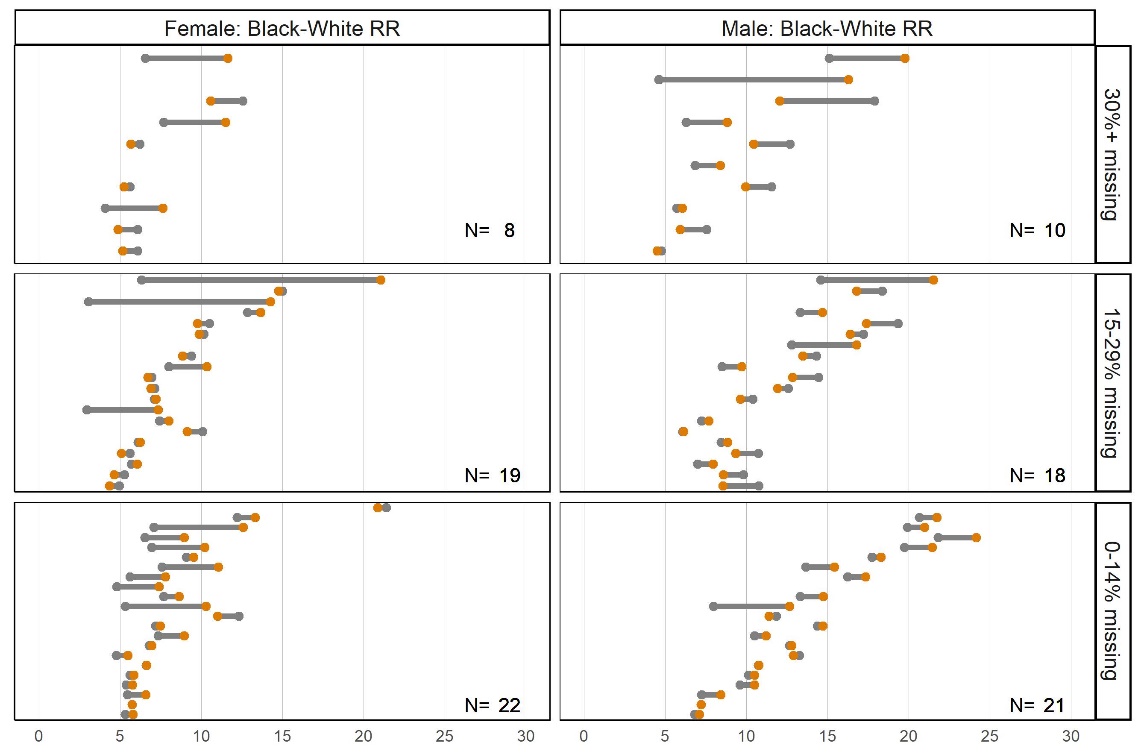 |
| --- |
| 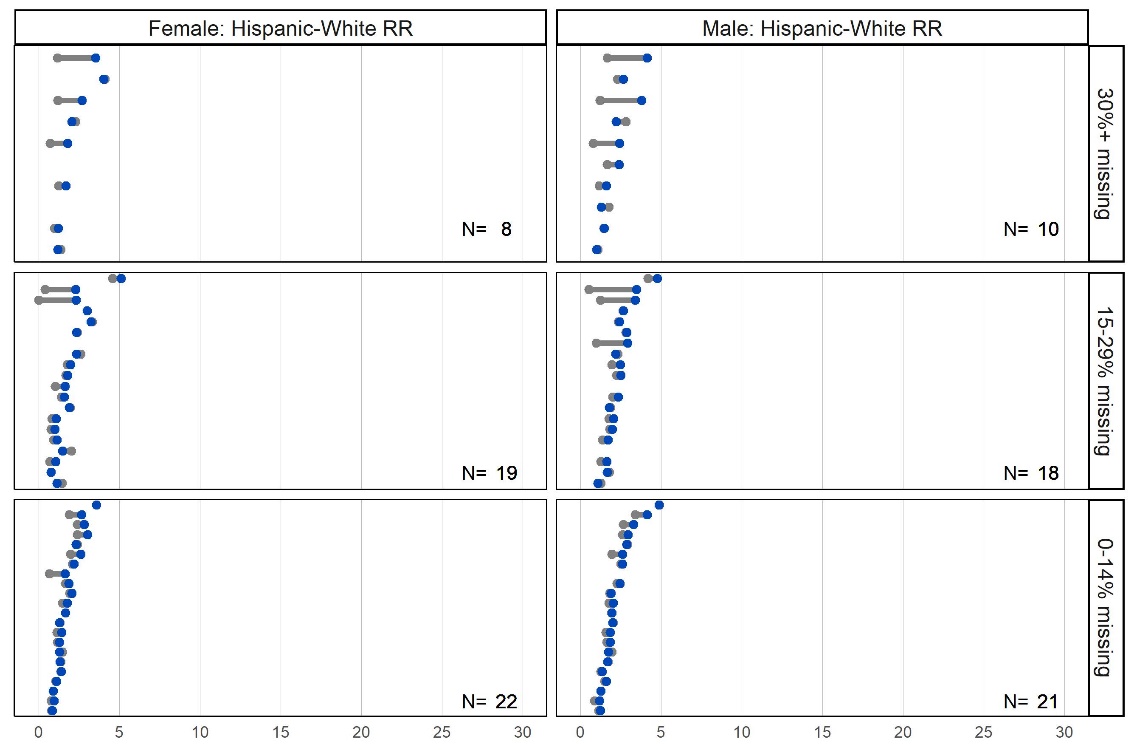 |

**Appendix Figure 17. Changes in rate differences for gonorrhea under the Scenario 4 compared to the base case**

| 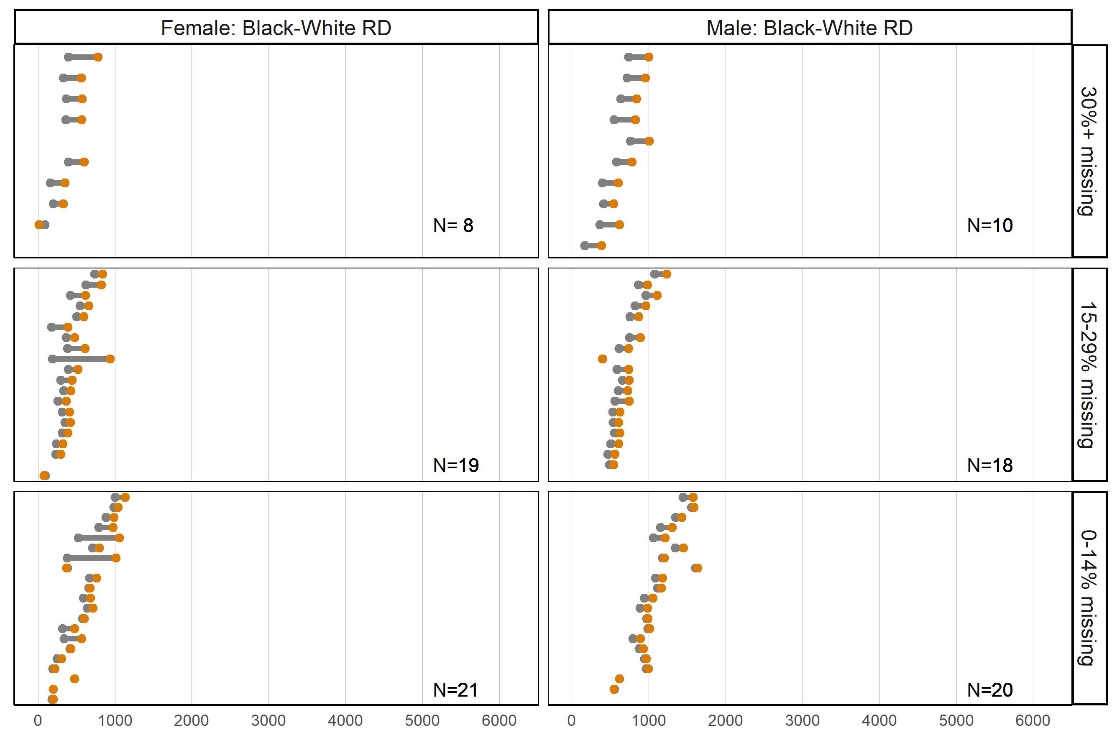 |
| --- |
| 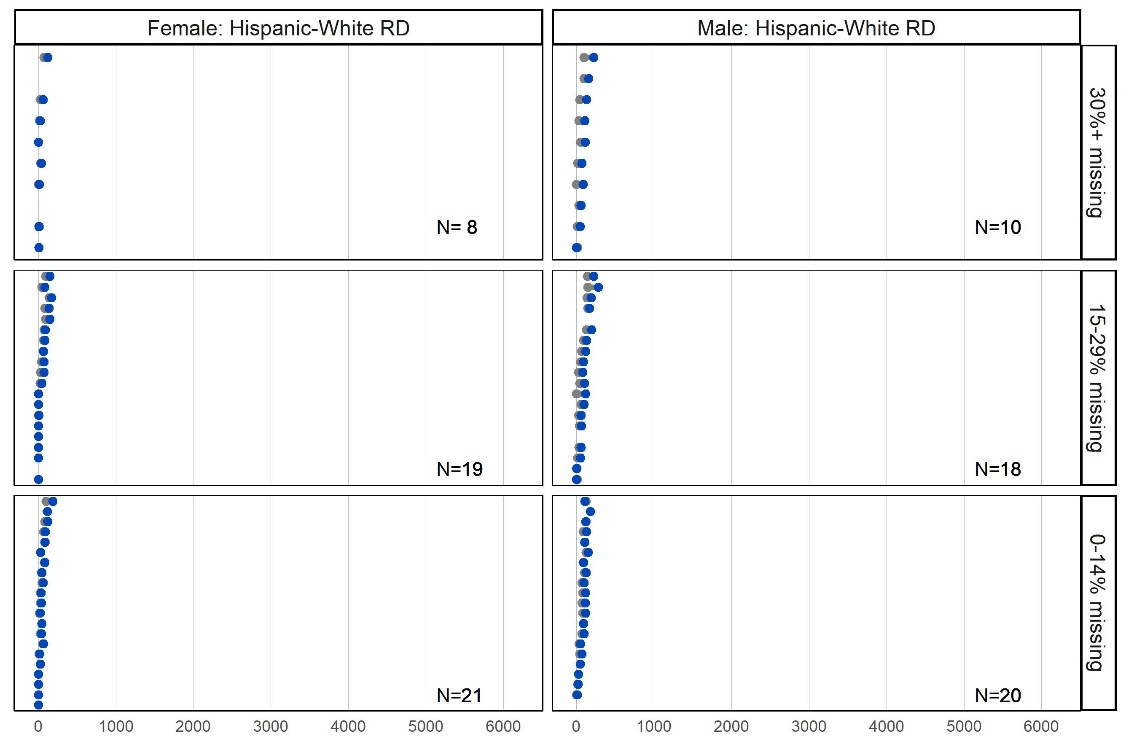 |

**Appendix Figure 18. Changes in relative rate ratios for gonorrhea under the Scenario 4 compared to the base case**

| 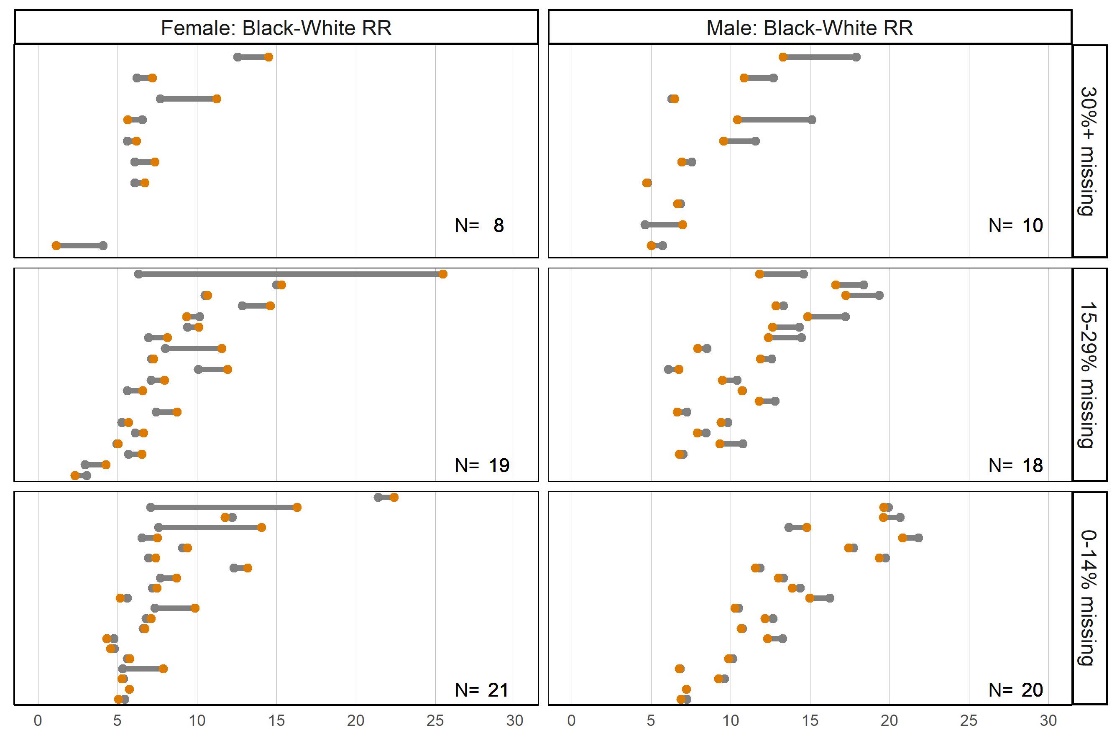 |
| --- |
| 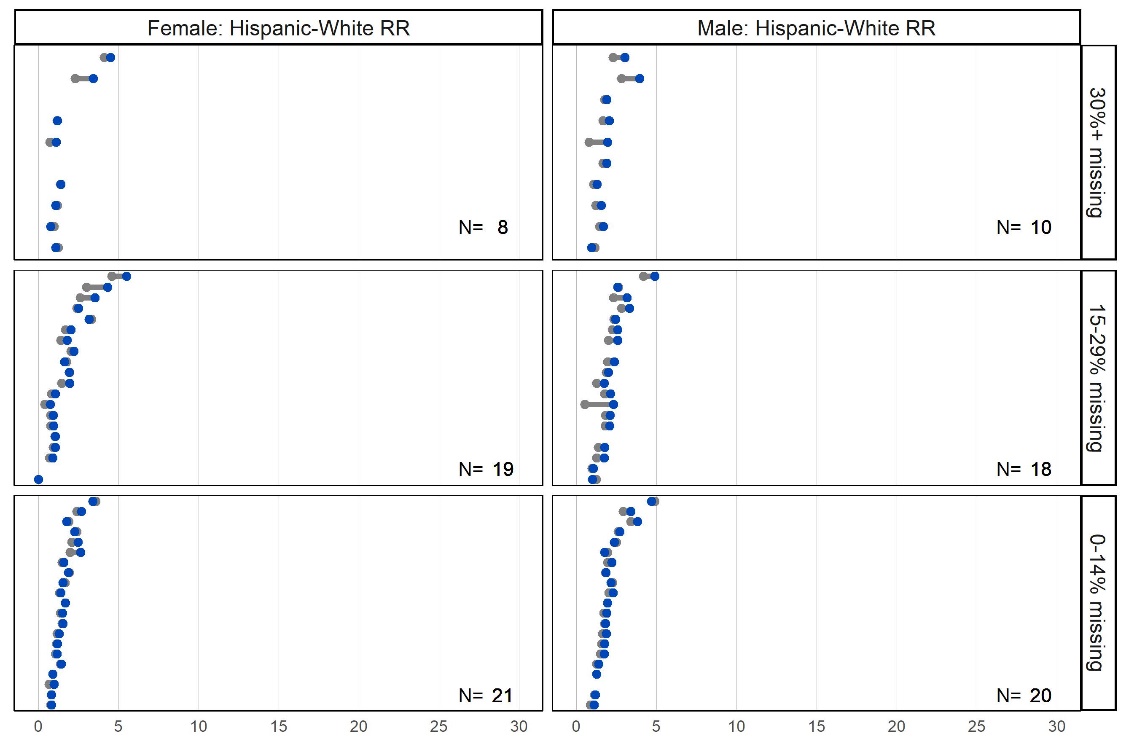 |

**Appendix Figure 19. Changes in rate differences for gonorrhea under the Scenario 5 compared to the base case**

| 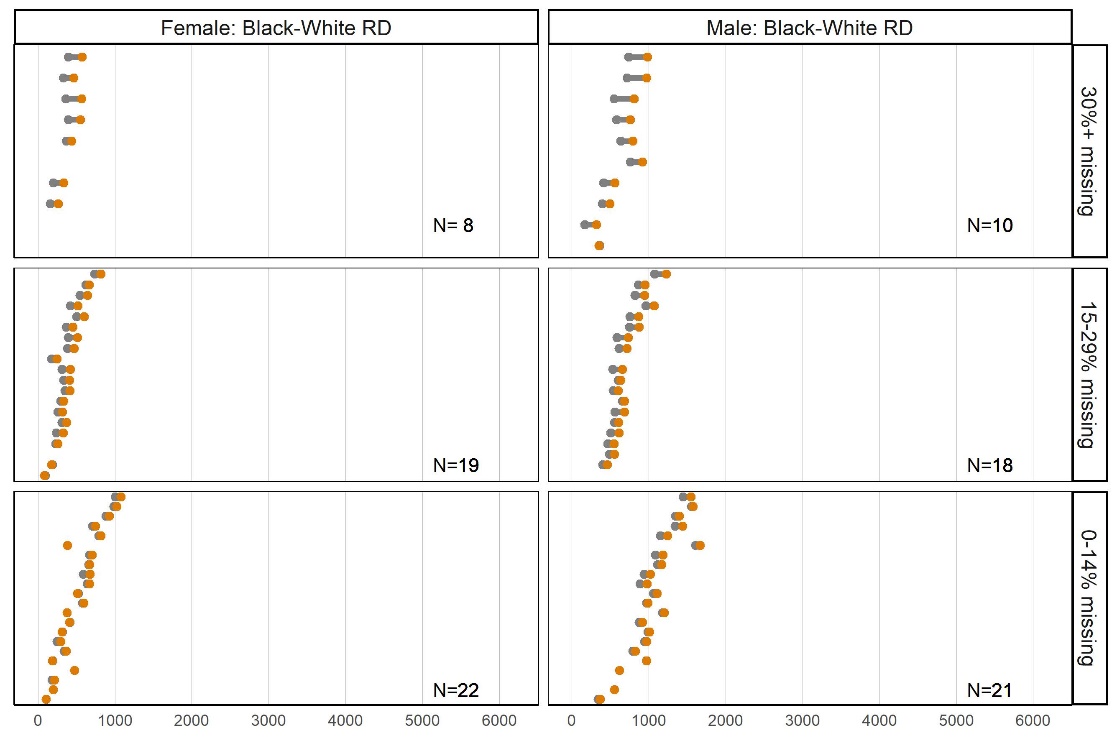 |
| --- |
| 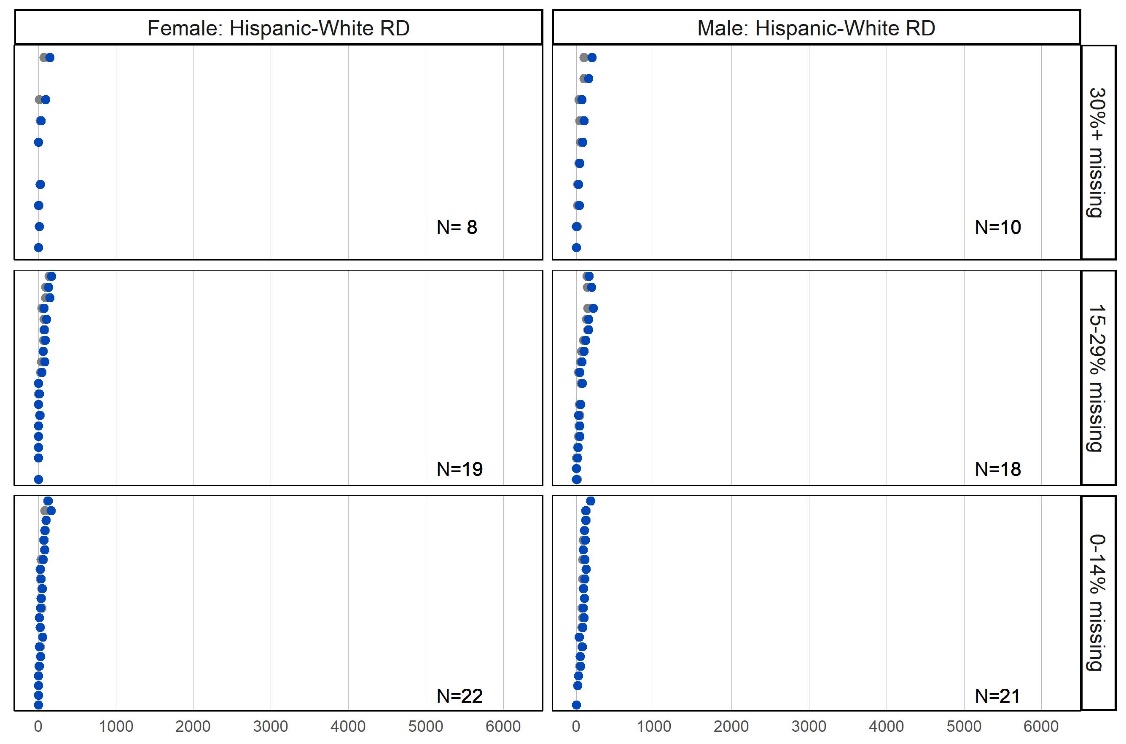 |

**Appendix Figure 20. Changes in relative rate ratios for gonorrhea under the Scenario 5 compared to the base case**

| 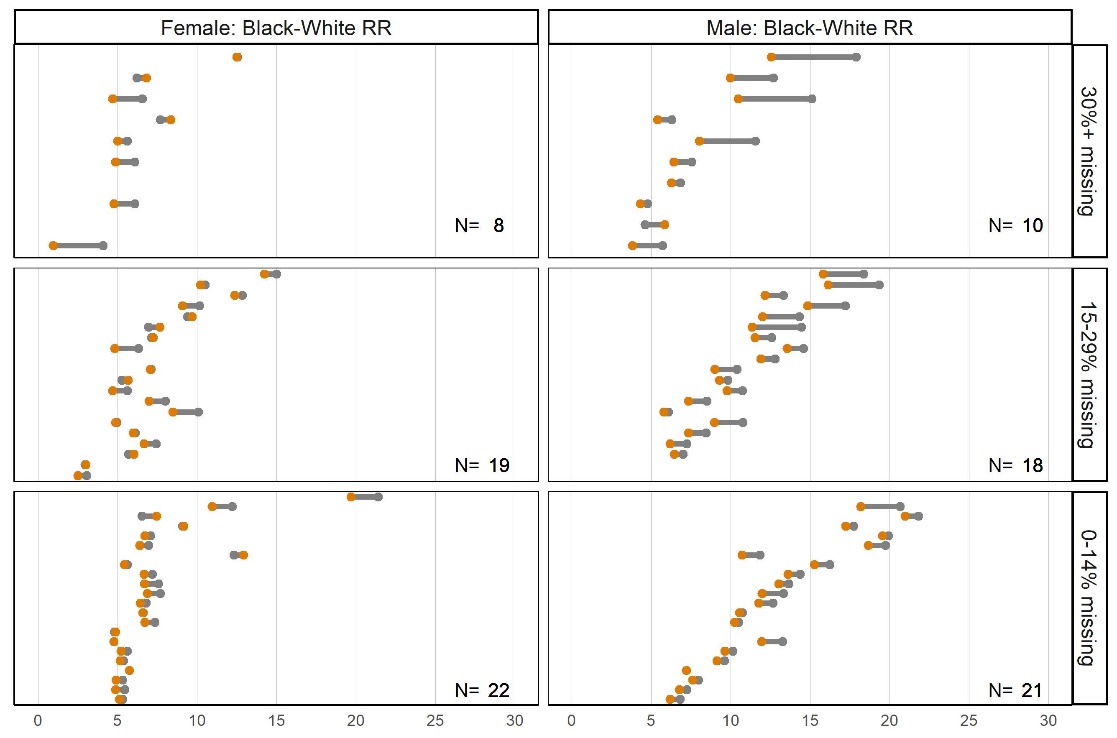 |
| --- |
| 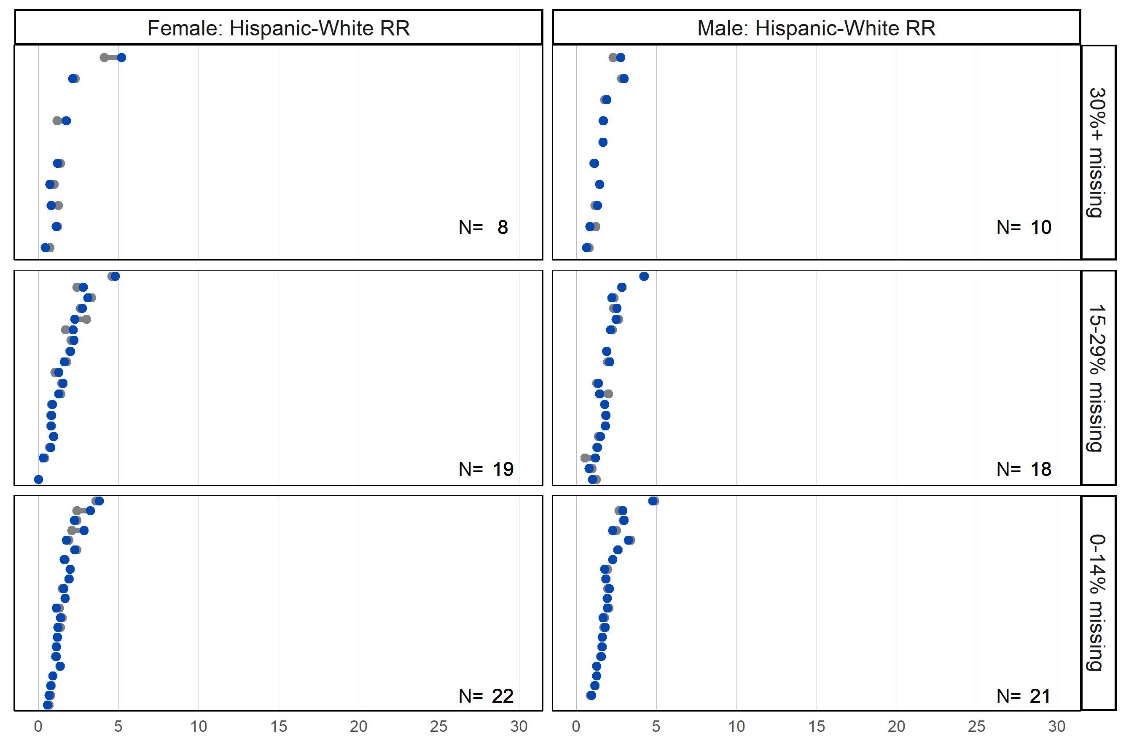 |
